# Low Memory T Cells Blood Counts and High Naïve Regulatory T Cells Percentage at Relapsing Remitting Multiple Sclerosis Diagnosis

**DOI:** 10.3389/fimmu.2022.901165

**Published:** 2022-05-30

**Authors:** João Canto-Gomes, Carolina S. Silva, Rita Rb-Silva, Daniela Boleixa, Ana Martins da Silva, Rémi Cheynier, Patrício Costa, Inés González-Suárez, Margarida Correia-Neves, João J. Cerqueira, Claudia Nobrega

**Affiliations:** ^1^ Life and Health Sciences Research Institute, School of Medicine, University of Minho, Braga, Portugal; ^2^ ICVS/3B’s, PT Government Associate Laboratory, Braga, Portugal; ^3^ Division of Infectious Diseases and Center for Molecular Medicine, Department of Medicine Solna, Karolinska Institutet, Stockholm, Sweden; ^4^ Department of Infectious Diseases, Karolinska University Hospital, Stockholm, Sweden; ^5^ Department of Onco-Hematology, Portuguese Institute of Oncology of Porto, Porto, Portugal; ^6^ Laboratory of Histology and Embryology, Department of Microscopy, ICBAS, University of Porto, Porto, Portugal; ^7^ Porto University Hospital Center, Porto, Portugal; ^8^ Multidisciplinary Unit for Biomedical Research (UMIB) - Instituto de Ciências Biomédicas Abel Salazar (ICBAS), University of Porto, Porto, Portugal; ^9^ Université Paris Cité, CNRS, INSERM, Institut Cochin, Paris, France; ^10^ University Hospital Complex of Vigo, Vigo, Spain; ^11^ Álvaro Cunqueiro Hospital, Vigo, Spain; ^12^ Hospital of Braga, Braga, Portugal; ^13^ Clinical Academic Centre, Hospital of Braga, Braga, Portugal

**Keywords:** multiple sclerosis, T cells, regulatory T cell (Treg), newly diagnosed, NK cell, treatment-naïve patients

## Abstract

**Objective:**

The aim of this study is to assess the peripheral immune system of newly diagnosed patients with relapsing remitting multiple sclerosis (RRMS) and compare it to healthy controls (HC).

**Methods:**

This cross-sectional study involves 30 treatment-naïve newly diagnosed patients with RRMS and 33 sex- and age-matched HC. Peripheral blood mononuclear cells were analyzed regarding: i) thymic function surrogates [T cell receptor excision circles (TRECs) and recent thymic emigrants (RTEs)]; ii) naïve and memory CD4^+^ and CD8^+^ T cells subsets; iii) T helper (Th) phenotype and chemokine receptors expression on CD8^+^ T cells subsets; iv) regulatory T cell (Tregs) phenotype; and exclude expression of activating/inhibitory receptors by natural killer (NK) and NKT cells. Analyses were controlled for age, sex, and human cytomegalovirus (HCMV) IgG seroprevalence.

**Results:**

Newly diagnosed patients with RRMS and HC have equivalent thymic function as determined by similar numbers of RTEs and levels of sjTRECs, DJβTRECs, and sj/DJβTREC ratio. In the CD8^+^ T cells compartment, patients with RRMS have a higher naive to memory ratio and lower memory cell counts in blood, specifically of effector memory and TemRA CD8^+^ T cells. Interestingly, higher numbers and percentages of central memory CD8^+^ T cells are associated with increasing time from the relapse. Among CD4^+^ T cells, lower blood counts of effector memory cells are found in patients upon controlling for sex, age, and anti-HCMV IgG seroprevalence. Higher numbers of CD4^+^ T cells (both naïve and memory) and of Th2 cells are associated with increasing time from the relapse; lower numbers of Th17 cells are associated with higher MS severity scores (MSSS). Patients with RRMS have a higher percentage of naïve Tregs compared with HC, and lower percentages of these cells are associated with higher MSSS. Percentages of immature CD56^bright^ NK cells expressing the inhibitory receptor KLRG1 and of mature CD56^dim^CD57^+^ NK cells expressing NKp30 are higher in patients. No major alterations are observed on NKT cells.

**Conclusion:**

Characterization of the peripheral immune system of treatment-naïve newly diagnosed patients with RRMS unveiled immune features present at clinical onset including lower memory T cells blood counts, particularly among CD8^+^ T cells, higher percentage of naïve Tregs and altered percentages of NK cells subsets expressing inhibitory or activating receptors. These findings might set the basis to better understand disease pathogenesis.

## Introduction

The immune system has the tricky task of fighting pathogens and tumor cells while maintaining tolerance to self, avoiding autoimmunity, and ensuring immune homeostasis. T cells are suggested to underlie multiple sclerosis (MS) pathogenesis most probably due to impaired tolerance to myelin-producing cells (1). MS is a demyelinating disease, clinically characterized by motor and cognitive impairment. The most common MS form (~85%), the relapsing-remitting MS (RRMS), is characterized by bouts of disability (called relapses) interleaved by periods of recovery (1).

In a mouse model of MS [experimental autoimmune encephalomyelitis (EAE)], peripheral administration of myelin-peptides triggers myelin-specific T cell responses in the central nervous system (CNS) and consequent demyelination (2). In humans, the relevance of T cells to MS pathophysiology is highlighted by the efficacy of MS therapies targeting T cells (3). Among the T cells involved in MS, the T helper (Th) 1 and Th17 cells have been proposed as key drivers of the immune attack against cells producing myelin peptides (4). Th cells subsets are characterized not only by their profile of cytokine production but also by the differential expression of chemokine receptors that confer them the migratory capacity to home to tissues (5). However, increasing evidence show the involvement of other T cells in MS, including the CD8^+^ T cells, which outnumber CD4^+^ T cells in MS lesions (6). In comparison to healthy controls (HC), patients with MS have been suggested to present lower thymic function and thus lower production of new T cells [recent thymic emigrants (RTEs)], and an aged peripheral adaptive immune system characterized by an accumulation of memory over naïve T cells (7–16).

Regulatory T cells (Tregs) are known for inducing tolerance by preventing activation of potential self-reactive T cells (17). Tregs have been extensively studied in MS yet observations on percentage, number, and function of these cells in MS are not consensual (7, 12, 13, 18–20). Several hypotheses on the ineffectiveness of Tregs in fully preventing MS have been proposed, including their limited capacity to access MS lesions in the CNS (in comparison to conventional T cells), ability of self-reactive T cells to withstand Tregs suppression, and lower number, percentage, and/or suppressive capacity of Tregs (21, 22). Both innate natural killer (NK) and NKT cells are also known to induce tolerance due to their killing potential of self-reactive T cells (23). NK cells’ activation and cytotoxicity toward anomalous cells is driven by the lack of engaging of inhibitory receptors and/or up-regulation and engaging of activating receptors to their specific ligands on target cells (24–26). NK cells from patients with MS were described to have reduced capacity to suppress autologous T cells proliferation *in vitro* in comparison to the ones from HC (23, 26). NKT cells establish a bridge between innate and adaptive immune system, presenting NK and T cell receptors (TCRs). In patients with MS, NKT cells were described to be hypo-responsive after stimulation with myelin-derived lipids (27).

Several immune system alterations have been described in patients with MS, however most of those studies lack data at clinical disease onset, before treatment initiation, which hampers the clarification of the immune features that are specific to MS and that might underlie its pathogenesis. Thus, the present study aims to provide an integrated evaluation of thymic function, peripheral T cell subsets homeostasis and of regulatory cells on treatment-naïve patients with RRMS at MS clinical onset.

## Results

### Thymic Export Is Unaltered in Newly Diagnosed Patients With RRMS

**Figure 1:**
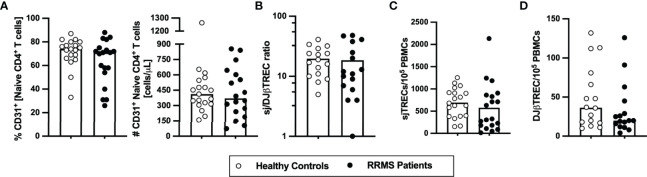
Thymic export in newly diagnosed RRMS patients does not differ from healthy controls. Percentage and number of CD31^+^ naïve CD4^+^ T cells **(A)**. Ratio of sj/DJβTRECs **(B)** calculated from the quantification of sjTRECs **(C)** and of DJβTRECs **(D)** levels per 10^5^ peripheral blood mononuclear cells (PBMCs). In all graphs, each dot represents one individual, and the horizontal lines represent the group’s mean or median, as the data follow a normal or non-normal distribution, respectively. Comparisons between healthy controls (white circles) and newly diagnosed RRMS patients (black circles) were performed using the t-test in **(B)** or the non-parametric Mann–Whitney *U*-test in **(A, C, D)**. Statistical outputs and effect size calculations in **eTable 1**. Results are maintained upon controlling for sex, age, and human cytomegalovirus IgG seroprevalence on multiple linear regression models (**eTable 2**).

To evaluate whether the thymic function, including intrathymic proliferation and thymic export, is altered in newly diagnosed patients with RRMS, two surrogates were quantified in the blood: i) percentage and number of RTEs, defined as CD31^+^ naïve CD4^+^ T cells; and ii) levels of TCR excision circles [TRECs; episomal DNA molecules formed upon TCR locus rearrangement; absolute quantification of sjTREC is a measure of thymic export, whereas the sj/DJβTRECs ratio is a measure for intrathymic proliferation (28)]. RTEs percentage and absolute numbers were unaltered in patients compared with controls (**Figure 1A**). No differences were found on the sj/DJβTRECs ratio nor on the sjTRECs and DJβTRECs levels (**Figures 1B–D**; **eTables 1, 2**). Overall, these results suggest that thymic function is not altered in newly diagnosed patients with RRMS.

### Newly Diagnosed RRMS Patients Have Lower Numbers of Circulating Memory T Cells

**Figure 2:**
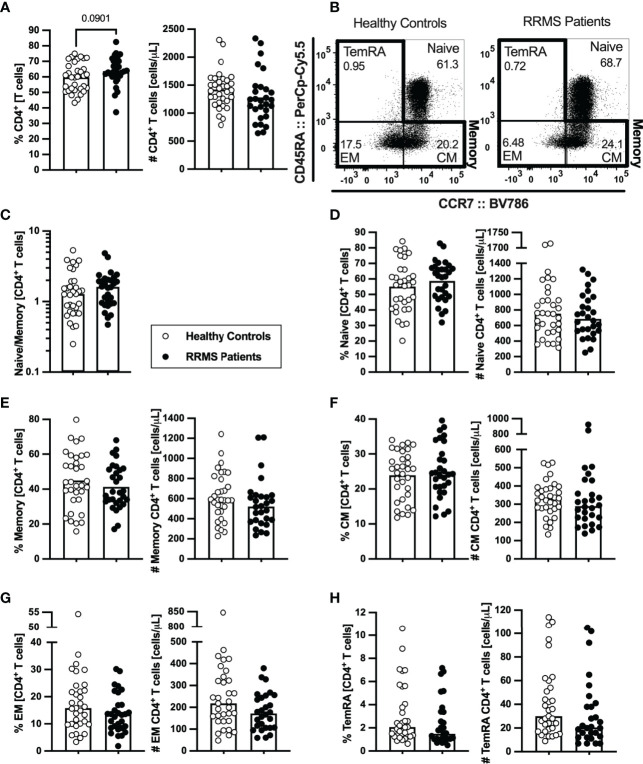
Newly diagnosed RRMS patients present no major differences on CD4^+^ T cells subsets. Percentages and numbers of CD4^+^ T cells are represented for healthy controls (white circles) and newly diagnosed RRMS patients [black circles; **(A)**]. Representative dot plots of the CD4^+^ T cell subpopulations **(B)**: naïve (CD45RA^+^CCR7^+^); central memory (CM; CD45RA^−^CCR7^+^), effector memory (EM; CD45RA^−^CCR7^-^); and terminally differentiated CD45RA-expressing memory T cells (TemRA; CD45RA^+^CCR7^−^). Total memory CD4^+^ T cells are the sum of the CM, EM, and TemRA subpopulations. Naïve to memory ratio of CD4^+^ T cells **(C)**. Percentages and numbers of CD4^+^ T cell subpopulations: naïve **(D)**, total memory **(E)**, central memory **(F)**, effector memory **(G)**, and TemRA **(H)**. The parametric Student’s *t-*test was performed in **(A)**, (**D–F**; percentage); the non-parametric Mann–Whitney *U*-test in **(C)**, (**D–F**; number), and **(G, H)**. Statistical outputs and effect size calculations in **eTable 1**. In all graphs, each dot represents one individual, and the horizontal lines the groups’ means or medians depending on the normal or non-normal distribution of the data, respectively. Results are maintained upon controlling for sex, age, and human cytomegalovirus (HCMV) IgG seroprevalence on multiple linear regression models (**eTable 3**), except for the percentage of CD4^+^ T cells **(A)**, whose tendency to be higher in patients is lost and for the effector memory CD4^+^ T cells whose absolute number becomes significantly lower in patients **(G)**.

As RTEs contribute to the peripheral seeding of the naïve T cell compartment and maintenance of naïve and memory T cells homeostasis (29), the circulating levels of naïve and memory CD4^+^ and CD8^+^ T cells were evaluated. The percentage and number of total CD4^+^ T cells did not differ between newly diagnosed patients with RRMS and HC (**Figure 2A**; **eTables 1, 3**). On the basis of CCR7 and CD45RA expression the naïve, central memory, effector memory, and terminally differentiated CD45RA-expressing memory T cells (TemRA) were defined (**Figure 2B**). Among CD4^+^ T cells, no differences were found on the naive to memory ratio (**Figure 2C**) nor on the percentages and numbers of naïve (**Figure 2D**), overall memory (**Figure 2E**), and central memory cells (**Figure 2F**). However, despite no differences are observed on the numbers and percentages of effector memory and TemRA CD4^+^ T cells upon direct comparison (**Figures 2G, H** and **eTable 1**), patients with RRMS present lower numbers of effector memory CD4^+^ T cells (**eTable 3**), when comparisons are adjusted for age, sex, and anti-HCMV IgG seroprevalence using multiple linear regression models. Regarding the Th phenotype, defined based on the differential expression of the chemokine receptors CCR4, CCR6, CCR10, and CxCR3 (5), no differences were found on the percentages or numbers of Th1, Th2, Th9, Th17, Th22, and GM-CSF-producing Th cells (ThG) (**Supplementary eFigure 1** and **eTables 1, 4**). In addition, no differences were found between groups regarding the percentage or number of the memory CD4^+^ T cells subsets expressing each of the chemokine receptors (data not shown).

**Figure 3:**
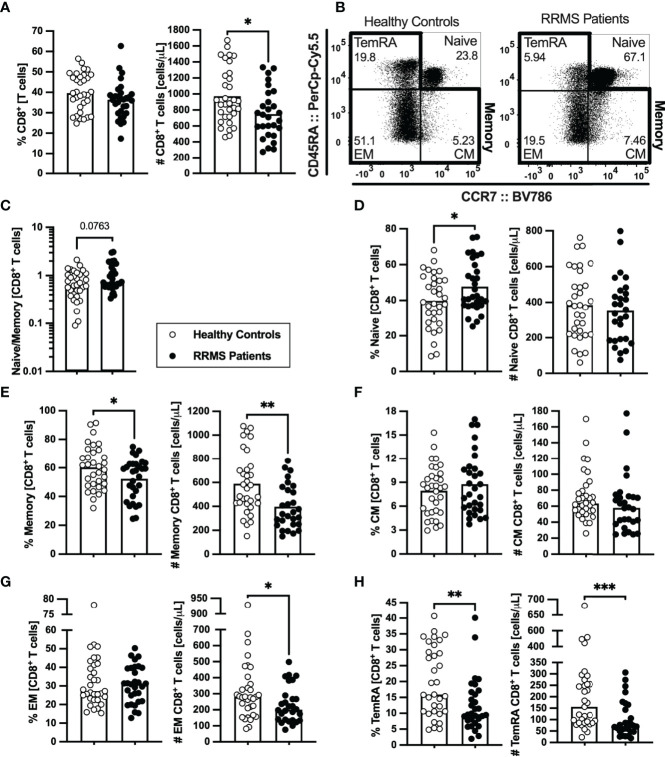
Newly diagnosed RRMS patients have lower numbers of memory CD8^+^ T cells and consequent higher naïve to memory ratio. Percentages and numbers of CD8^+^ T cells are represented for healthy controls (white circles) and newly diagnosed RRMS patients (black circles; **(A)**. Representative dot plots of the CD8^+^ T cell subpopulations **(B)**: naïve (CD45RA^+^CCR7^+^); central memory (CM; CD45RA^-^CCR7^+^), effector memory (EM; CD45RA^-^CCR7^-^); and terminally differentiated CD45RA-expressing memory T cells [TemRA; CD45RA^+^CCR7^−^). Total memory CD8^+^ T cells are the sum of the CM, EM, and TemRA subpopulations. Naïve to memory ratio of CD8^+^ T cells **(C)**. Percentages and numbers of CD8^+^ T cell subpopulations: naïve **(D)**, total memory **(E)**, central memory **(F)**, effector memory **(G)**, and TemRA **(H)**. The parametric Student’s *t-*test was performed in **(A**; number), **(D, E)**, (**F**; percentage); the non-parametric Mann–Whitney *U*-test in **(A**; percentage), **(C)**, (**F**; number), and **(G, H)**. Statistical outputs and effect size calculations in **eTable 1**. In all graphs, each dot represents one individual, and the horizontal lines represent the groups’ means or medians depending on the normal or non-normal distribution of the data, respectively. When differences were statistically significant (*p-value* < 0.050), the *p*-value was represented by * for 0.010 < *p* < 0.050; **0.001 < *p ≤* 0.010; and ***p ≤ 0.001. Results are maintained upon controlling for sex, age, and human cytomegalovirus IgG seroprevalence on multiple linear regression models (**eTable 5**).

Lower numbers of CD8^+^ T cells were found in patients with RRMS (**Figure 3A**; **eTables 1, 5**). Concerning the naïve and memory subsets (**Figure 3B**), patients presented a tendency to lower naïve to memory CD8^+^ T cells’ ratio that was found to be associated with the disease in the multiple linear regression model (**Figure 3C**; **eTables 1, 5**). Although no differences were observed on the number of naïve CD8^+^ T cells (**Figure 3D**; **eTables 1, 5**), patients with RRMS present lower percentages and numbers of memory cells (**Figure 3E**; **eTables 1, 5**). Regarding the memory CD8^+^ T cell subsets (**Figures 3B, F–H**; **eTables 1, 5**), lower numbers of effector memory and TemRA cells were observed in patients (**Figures 3G, H**). We also found differences on CD8^+^ T cells expressing chemokine receptors, specifically the CCR6 (**Supplementary eFigures 2A–D**). Patients with RRMS presented lower percentage and number of TemRA CD8^+^ T cells and lower number of effector memory CD8^+^ T cells expressing CCR6 (**Supplementary eFigures 2A–D**; **eTable 1**), however the statistical significance of the latter was lost upon controlling for age, sex, and anti-HCMV IgG seroprevalence in the multiple linear regression model (**eTable 6**).

In summary, newly diagnosed patients with RRMS present a general contraction of the peripheral memory T cell compartment, particularly pronounced in the CD8^+^ T cell subset.

### Newly Diagnosed RRMS Patients Have Higher Percentages of Naïve Regulatory T Cells

**Figure 4:**
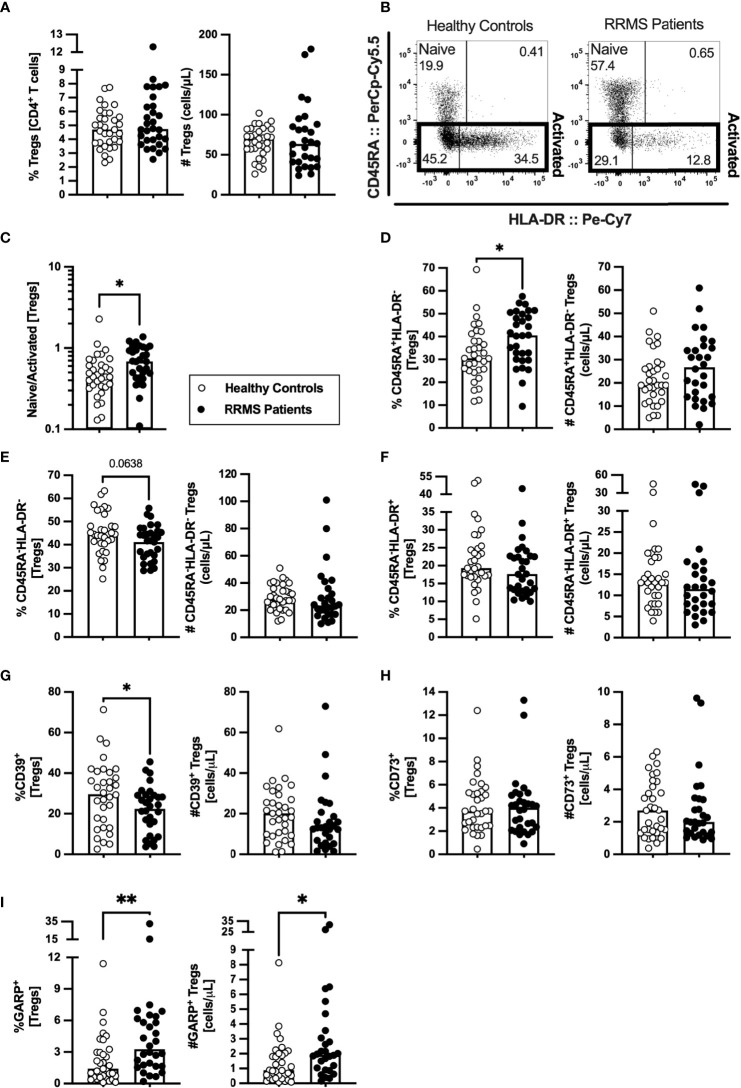
A higher naïve to activated Tregs’ ratio characterize newly diagnosed RRMS patients. Percentage and number of Tregs are represented for healthy controls (white circles) and newly diagnosed RRMS patients [black circles; **(A)**]. Representative dot plot of Tregs subpopulations **(B)**: naïve (CD45RA^+^HLA-DR^−^); activated HLA-DR^−^ (CD45RA^−^HLA-DR^−^); and activated HLA-DR^+^ (CD45RA^−^HLA-DR^+^). Total activated Tregs are the sum of the HLA-DR^−^ and HLA-DR^+^ subpopulations. Naïve to activated Tregs’ ratio **(C)**. Percentages and numbers of Tregs subpopulations: naïve **(D)**, activated HLA-DR^−^ **(E)**, and activated HLA-DR^+^ **(F)**. Percentages and numbers of Tregs expressing suppressive markers: CD39^+^ **(G)**, CD73^+^ **(H)**, and GARP^+^ **(I)**. The parametric Student’s *t-*test was performed in **(D**; number), **(E)**; percentage), and **(G**; (percentage); the non-parametric Mann–Whitney *U*-test in **(A, C)**, (**D**; percentage), (**E**; number), **(F)**, (**G**; number), and **(H, I)**. Statistical outputs and effect size calculations in **eTable 1**. In all graphs, each dot represents one individual, and the horizontal lines the groups’ means or medians depending on the normal or non-normal distribution of the data, respectively. When differences were statistically significant (*p-value <*0.050), the *p*-value was represented by * for 0.010< *p* <0.050 and ** 0.001< *p ≤*0.010. Differences are maintained upon controlling for sex, age, and human cytomegalovirus IgG seroprevalence on multiple linear regression models (**eTable 7**), except for the naïve/activated Tregs’ ratio that becomes a tendency to be higher on patients with RRMS **(C)**, and the percentage of activated HLA-DR^+^ Tregs that becomes a tendency to be lower in patients **(F)**.

Tregs are scarcely characterized at MS clinical onset, and their role on MS pathogenesis is yet unknown. In newly diagnosed patients with RRMS, no differences were found on the percentage or absolute number of Tregs compared with HC (**Figure 4A**; **eTables 1, 7**). HLA-DR expression level is described to positively correlate with FOXP3 expression and with Tregs suppressive capacity (30). Three subsets of Tregs were defined based on HLA-DR and CD45RA expression: the naïve (CD45RA^+^HLA-DR^−^), and the activated HLA-DR^−^ (CD45RA^−^HLA-DR^−^) and HLA-DR^+^ (CD45RA^−^HLA-DR^+^) (**Figure 4B**). Higher naive to activated Tregs' ratio was observed in patients (**Figure 4C**). Regarding naïve Tregs, patients presented higher percentages of these cells, but no differences in numbers (**Figure 4D**). A tendency to a lower percentage of activated HLA-DR^−^ Tregs was found in patients (**Figure 4E**; also observed in the regression models, **eTable 7**). No differences were found on activated HLA-DR^+^ Tregs (**Figure 4F**) although, upon controlling for age, sex, and anti-HCMV IgG seroprevalence, newly diagnosed patients with RRMS exhibited a tendency to lower percentage of activated HLA-DR^+^ Tregs (**eTable 7**).

The suppressive Treg markers CD39, CD73, and GARP were also evaluated to assess Tregs suppressive potential. Newly diagnosed patients with RRMS presented a lower percentage of CD39^+^ Tregs (**Figure 4G**) and no differences on CD73^+^ Tregs (**Figure 4H**). A higher percentage and number of Tregs expressing GARP was observed in patients (**Figure 4I**). Notwithstanding, the multiple linear regression model for the number of GARP-expressing Tregs was not statistically significant (**eTable 7**).

These results point toward newly diagnosed patients with RRMS having Tregs with a more naïve phenotype than HC.

### RRMS Patients at Clinical Diagnosis Have Higher Percentages of CD56^bright^ NK Cells Expressing KLRG1^+^ and of CD56^dim^CD57^+^ NK Cells Expressing NKp30^+^

**Figure 5:**
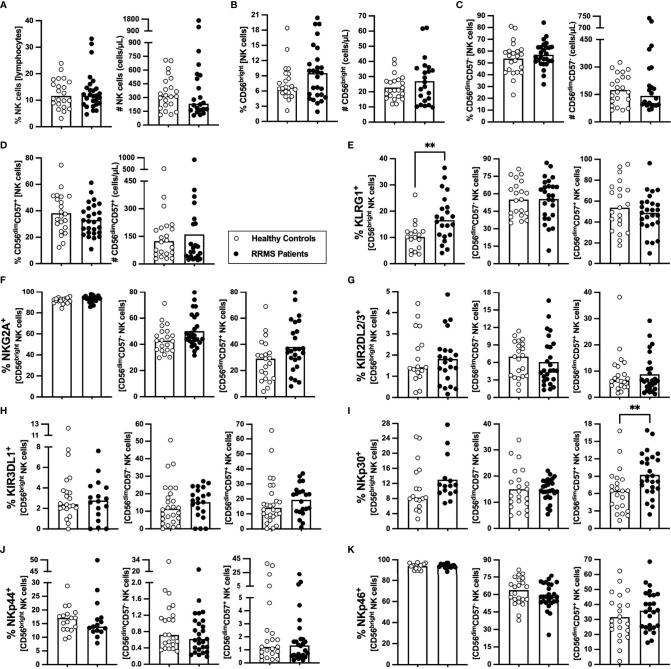
Newly diagnosed RRMS patients have higher percentages of CD56^bright^ NK cells expressing KLRG1, and CD56^dim^CD57^+^ expressing NKp30. Percentages and numbers of NK are represented for healthy controls (white circles) and newly diagnosed RRMS patients [black circles; **(A)**]. Percentages and numbers of NK cells subpopulations: CD56^bright^ [most immature; **(B)**]; CD56^dim^CD57^−^ **(C)**; and CD56^dim^CD57^+^ (most differentiated; **(D)**). Percentage of cells expressing inhibitory receptors in the three NK cells subsets: KLRG1 **(E)**, NKG2A **(F)**, KIR2DL2/3 **(G)** and KIR3DL1 **(H)**. Percentage of cells expressing activating receptors in the three NK cells subsets: NKp30 **(I)**, NKp44 **(J)**, and NKp46 **(K)**. The parametric Student’s *t-*test was performed in **(B**; number), (**C, D**; percentage), **(E**; CD56^dim^CD57^−^ and CD56^dim^CD57^+^), **(F**; CD56^dim^CD57^−^ and CD56^dim^CD57^+^), **(G**; CD56^bright^ and CD56^dim^CD57^−^), **I**; CD56^dim^CD57^−^), and **K**; CD56^bright^ and CD56^dim^CD57^+^); the non-parametric Mann–Whitney *U*-test in **(A)**, (**B**; percentage), (**C, D**; number), **(E)**; CD56^bright^), **(F)**; CD56^bright^), **(G)**; CD56^dim^CD57^+^), **(H)**, (**I**; CD56^bright^ and CD56^dim^CD57^+^), **(J)**, and (**K**; CD56dimCD57^-^). Statistical outputs and effect size calculations in **eTable 1**. In all graphs, each dot represents one individual, and the horizontal lines the groups’ means or medians depending on the normal or non-normal distribution of the data, respectively. When differences were statistically significant (*p-value <*0.050), the *p*-value was represented by ** 0.001< *p ≤*0.010. Results are maintained upon controlling for sex, age, and human cytomegalovirus IgG seroprevalence on multiple linear regression models (**eTable 8**).

NK cells characterization at clinical RRMS onset is poorly explored. At clinical diagnosis, patients with RRMS were not different from HC on the percentage and number of NK cells (**Figure 5A**) or of any of its subsets [CD56^bright^ (most immature), CD56^dim^CD57^−^, and CD56^dim^CD57^+^ (most differentiated); **Figures 5B–D**)]. Newly diagnosed patients with RRMS had higher percentage of KLRG1^+^ cells among CD56^bright^ NK cells (**Figure 5E**) but no differences on the percentage of cells expressing any of the other inhibitory receptors evaluated, such as NKG2A, KIR2DL2/3, and KIRDL1 (**Figures 5F–H**; **eTables 1, 8**). Regarding the expression of the activating receptors, a higher percentage of cells expressing NKp30^+^ cells was found among CD56^dim^CD57^+^ NK cells in patients (**Figure 5I**). No differences were observed on the percentage of cells expressing NKp44 or NKp46 receptors for any of the NK cell subsets (**Figures 5J, K**; **eTables 1, 8**).

Newly diagnosed patients with RRMS present no major differences on NKT cells concerning percentages and numbers (**Supplementary eFigure 3A**). No differences were observed on the percentage of NKT cells expressing inhibitory/activating receptors (**Supplementary eFigures 3B–H**). The lower percentages of NKT cells expressing KIR3DL1 (**Supplementary eFigure 3E**) was found to be related to anti-HCMV IgG seroprevalence and not with the disease (**eTable 9**).

These results show that NK cells from newly diagnosed patients with RRMS have increased percentages of the inhibitory KLRG1^+^ within the most immature NK cells subset and increased percentages of the activating NKp30^+^ within the most mature NK cells subset.

### Time From the Relapse and MS Severity Score Correlate With Alterations in Immune Cell Populations

**Figure 6:**
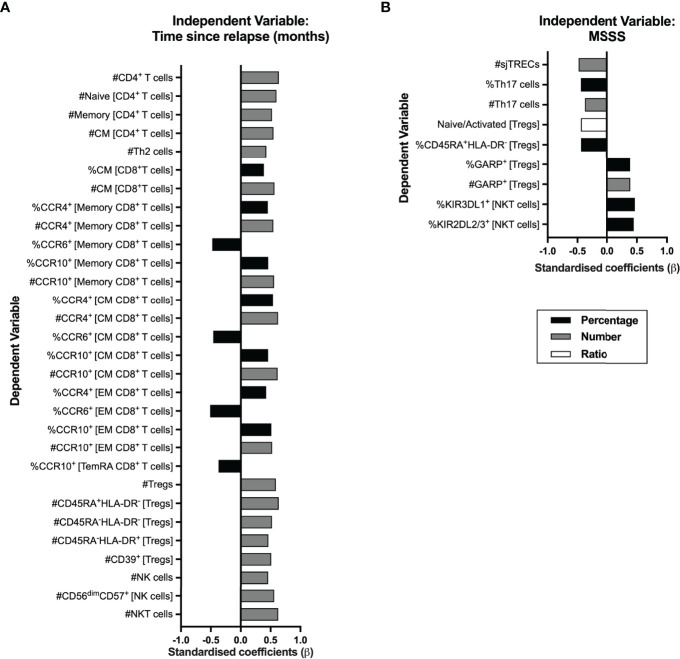
Time from the exclude relapse and MS severity score (MSSS) relates to immune system cell alterations in RRMS patients. Represented are the standardized coefficients (β) from multiple or linear regression models having as exclude independent variable the time from the exclude relapse **(A)** or the MSSS **(B)**, respectively. The dependent variable consisted of the unstandardized residuals from a preliminary multiple linear regression where the contribution of sex, age, and anti-HCMV IgG seroprevalence on the blood populations was evaluated (as described in the Materials and Methods section). Only the blood populations for which the linear regression models were significant (*p <*0.050) for the variable of interest are represented. Statistical outputs and effect size calculations are represented in **eTables 10, 11**.

In RRMS, a relapse is a clinical manifestation of disease activity and results from an immune reaction that drives myelin destruction, and consequent neuronal damage and impairment (1). All patients from this cohort were on remission at the time of the immune characterization, although the vast majority (78%; 21 patients) had a relapse within the 10 months prior sampling. We analyzed whether the time from the relapse was correlated with peripheral immune alterations. As some patients had a corticosteroid pulse to treat the relapse, this was included as a dichotomous variable in the regression models evaluating the contribution of the time from the relapse to immune cells alterations to control for its possible confounding effect (**eTable 10**). Alterations on CD4^+^ and CD8^+^ T cells, Tregs, NK cells, and NKT cells subsets were found to be related with time from the relapse (**Figure 6A**; **eTable 10**). The increasing time from the relapse was associated with higher numbers of total CD4^+^ T cells, both naïve and total memory (specifically central memory and Th2 cells). Regarding CD8^+^ T cells, higher percentage and number of central memory cells was related with increasing time from the relapse. In addition, increasing time from the relapse was correlated with increased numbers and percentages of CCR10^+^ and CCR4^+^ cells among total, central, and effector memory CD8^+^ T cells, and with a decrease in the percentages of CCR6^+^ cells in those cell subsets.

Increasing numbers of total Tregs and of its subsets based on HLA-DR and CD45RA and of Tregs expressing CD39 were associated with increasing time from the relapse. Higher numbers of total and of CD56^dim^CD57^+^ (the most differentiated NK subset) NK cells and of NKT cells correlated with increasing time from the relapse.

We also investigated a possible association between the MS Severity Score and the alterations on blood cell populations (**Figure 6B**; **eTable 11**). A higher MSSS correlated with lower sjTRECs levels and lower percentages and numbers of Th17 cells. Lower naïve/activated Tregs’ ratio and percentage of naïve Tregs (CD45RA^+^HLA-DR^−^) correlated with increasing MSSS. Higher percentage and number of Tregs expressing GARP was associated with higher MSSS. On NKT cells, higher MSSS correlated with higher percentage of cells expressing the KIR3DL1 and KIR2DL2/3 inhibitory receptors. No association was found between MS severity and CD8^+^ T cells subsets.

## Discussion

Several reports have evaluated thymic function and blood cell populations in patients with MS (7–15). Most of those studies described an aged immune system in patients with MS in comparison to HC as assessed by thymic function, naïve to memory T cells’ ratio, among other parameters. However, in some of those studies, the group of patients with MS is quite heterogeneous, combining in the same set patients with different MS progression forms (7, 15), diverse disease durations (10) or patients with RRMS on relapse with patients on remission (11). In addition, in some instances, information regarding the MS treatment history is missing which hampers our comprehension on whether the effect of immunotherapies on immune cell populations was considered or neglected (15). Here, we based our study on a homogenous group of newly diagnosed patients with RRMS (<17 months [median of 1 month] after disease diagnosis), naïve for disease modifying drugs and recruited from three distinct hospitals. The study of these newly diagnosed patients with RRMS and of age- and sex-matched HC allowed us to provide an integrative view of thymic function and of the several blood immune cell populations at MS clinical onset. In addition, it adds on the relation between immune alterations and time from the relapse and MS severity. All these observations were controlled for age, sex, and anti-HCMV IgG seroprevalence, which are variables known to affect the percentages and numbers of blood cell populations (20, 31).

We report here similar thymic function on newly diagnosed patients with RRMS and HC upon controlling for age, sex, and anti-HCMV IgG seroprevalence, as observed by equivalent levels of RTEs, sjTRECs, DJβTRECs, and sj/DJβTREC ratio. Despite many studies support a lower thymic function in patients with RRMS (8, 11–13), at least one other report corroborates our data on untreated patients with RRMS (14). Moreover, in a cohort of treatment-naïve monozygotic twins discordant for MS, it was found that RRMS itself was not associated with RTEs alterations (32). We found that higher MSSS are related to lower sjTRECs levels in patients, but not with the sjTREC/DJβTREC ratio. Unlike sjTREC/DJβTREC ratio, sjTRECs quantification, by itself, is not a good surrogate of thymic function as cells containing sjTRECs get diluted with cell proliferation (28). For this reason, the alteration on sjTRECs with MSSS should not be related with altered thymic function.

When comparing the median number of effector memory CD4^+^ T cells, no differences were observed between HC and patients with RRMS. However, in the multiple linear regression model, upon controlling for age, sex, and anti-HCMV IgG seroprevalence, we observed that newly diagnosed patients with RRMS present lower number of effector memory CD4^+^ T cells (B = −68.94 cells/μl, being the reference the HC). This difference between the results obtained from the direct comparison of the medians vs. the multiple linear regression model might be due to the fact that, with age, the number of effector memory CD4^+^ T cells increases (9.96 cells/μl/year), as previously described (29, 33). In this case, sex or anti-HCMV IgG seroprevalence had no considerable contribution to alterations in the abovementioned cell subset. A previous study reported that untreated patients with RRMS had lower percentage, and a tendency to lower number, of early effector memory CD4^+^ T cells and speculated that those alterations were attributed to T-cell migration into the CNS (14). Curiously, another study observed a cluster of central/effector memory T cells with a CNS homing signature that was reduced in the blood of patients with MS and enriched in lesions of post-mortem brain tissue of patients with MS, in comparison to HC (34). The magnitude of the observed differences suggests a systemic alteration on CD4^+^ T cells, and not specifically on myelin specific CD4^+^ T cells. In fact, those cells represent a negligible population of approximately 0.0001% of all CD4^+^ T cells and their percentage in blood has been described to not differ between RRMS patients and HC (35). Notwithstanding, the fact that no differences are described regarding the number and/or percentage of myelin-reactive T cells does not necessarily mean that the function and reactivity of those cells is similar between HC and RRMS individuals. exclude, Greer et al. have shown that T cells from patients with RRMS and secondary progressive (SP) MS are more reactive to some myelin peptides, namely, the myelin proteolytic protein (PLP), than T cells from HC (36). Interestingly, this higher reactivity to PLP was more evident in patients with 6–15 years of disease duration and with moderate to severe disability; T cell reactivity to PLP was not different between newly diagnosed patients and HC. These data seem to suggest that the higher reactivity of T cells to PLP is not evident at MS pathogenesis; it might although be a consequence of posterior extensive myelin damage. Interestingly, the reactivity to myelin peptides was higher in females than in males (37). From our data, we cannot infer on whether the lower number of effector memory CD4^+^ T cells is related to altered proneness to migrate to tissues as no major differences were found between groups on the expression of chemokine receptors, nor on any of the other memory CD4^+^ T cell subsets (data not shown).

As time from the relapse increases, higher numbers of blood CD4^+^ T cells were observed, particularly of the naïve and central memory cells, pointing toward a possible involvement of these cells on MS relapses, although the link between naïve and central memory CD4^+^ T cells reduction and MS pathophysiology was not explored.

Th1 and Th17 cells have been suggested to be involved in MS pathophysiology (4). Newly diagnosed patients with RRMS did not differ from HC on the percentage or number of Th1 and Th17, nor on the balance of Th17/Tregs (data not shown), whose imbalance has been implicated in autoimmunity (38). Still, we observed a correlation between lower percentage and number of Th17 cells and higher MSSS; whether this is due to migration and accumulation of those cells on the CNS of patients and whether that impacts disease severity must be further explored.

It is known that MS is more prevalent among women and, in line with this, that some populations were shown to be increased (e.g., Th1 cells) or decreased (e.g., Th2 cells) in women living with MS (39–41). We observe that sex has an impact in Th2 percentages and numbers, and in other cell populations, upon adjusting for age, anti-HCMV IgG seroprevalence, and disease in the multiple linear regression models. However, in these models, it is not possible to infer whether that sex-effect is observed for women with RRMS and/or HC. For this, a sex-stratified analysis would be required, which is not possible in our cohort given its limited sample size.

We observed lower numbers of CD8^+^ T cells in the blood of patients with RRMS. CD8^+^ T cells are known to surpass CD4^+^ T cells in MS lesions, which may imply a higher recruitment of CD8^+^ T cells to the CNS (6). Our data show decreased numbers of CCR6-expressing memory CD8^+^ T cells in patients, which might be related to increased migration of these cells to tissues. Interestingly, CCR6 has been described to facilitate brain-homing migration and to be essential for EAE induction (42). In addition, the choroid plexus constitutively expresses the CCR6-ligand CCL20 and constitutes the most probable route of entry for those cells into the CNS (42). Interestingly, lower percentages of memory CD8^+^ T cells expressing CCR6 were associated with increasing time from the relapse, suggesting that during a relapse a higher percentage of cells bearing brain-homing potential might be present in circulation. We observed that patients with RRMS present higher naive to memory ratio among CD8^+^ T cells and lower number of memory T cells, specifically of effector memory and TemRA. Lower percentage of these memory CD8^+^ T cells subsets was previously observed in a group of patients with untreated RRMS and clinically isolated syndrome, in comparison to HC (43). The authors attributed the lower effector memory and TemRA CD8^+^ T cells to primary intrinsic defects (genetically determined) rather than being a pathophisiological consequence of MS. In addition, the authors claim that the memory CD8^+^ T cell deficiency is present at MS onset and persists throughout its course independently of disease severity, disability, or duration (43). Curiously, CD8^+^ T cells deficiency is characteristic of many other chronic autoimmune disorders (e.g., rheumatoid arthritis, systemic lupus erythematosus, Crohn’s disease, type 1 diabetes mellitus, and myasthenia gravis) and is also found in the blood of patients’ blood relatives (44). This supports that CD8^+^ T cells deficiency might be inherited and underly autoimmunity.

The essential role of Tregs to immune tolerance highlights the importance of these cells in autoimmune disorders (17). Our data show that in comparison to HC, newly diagnosed patients with RRMS present higher percentages of naïve Tregs and tendency to lower percentages of activated HLA-DR^+^ and HLA-DR^−^ Tregs. These results contrast with previous findings showing higher percentage of activated HLA-DR^+^ and lower of naïve Tregs in patients with MS (19, 45). Our focus specifically on newly diagnosed patients with RRMS and the combination of CD25, CD127, and FOXP3 expression to define Tregs (and its subsets using HLA-DR and CD45RA) might have directed us to these observations. The percentage of cells expressing the suppressive marker CD39 was lower in patients’ Tregs, which might be related to the fact that this marker was mostly expressed on activated Tregs that were also tendentially lower in patients with RRMS. In fact, the percentage of CD39^+^ among activated Tregs was not different between groups (data not shown), thus suggesting that the suppressive capacity of activated Tregs is not altered, as assessed by CD39. On the other hand, the percentage and number of GARP-expressing Tregs is higher in patients with RRMS, which is suggestive of a higher proportion of suppressive Tregs in patients. Similar findings were previously made in patients with RRMS, and the authors attributed it to systemic inflammation rather than being a functional defect of patients’ Tregs (46). A higher number of Tregs (and of all its subsets based on CD45RA and HLA-DR expression), and of Tregs expressing CD39 was correlated with increasing time from the relapse. On the other hand, increasing MSSS was linked with decreased naïve/activated ratio and percentage of naïve Tregs, and with higher numbers of suppressive GARP^+^ Tregs. Together, these results suggest that during a relapse fewer Tregs, including Tregs bearing suppressive potential through CD39 expression, are present in the blood of patients and that patients with higher MSSS have more Tregs expressing GARP and lower percentage of naïve Tregs. Although, a longitudinal study evaluating these cell subsets in treatment naive RRMS patients on relapse and in remission would be valuable to clarify these aspects. Notwithstanding, our results correlate with previous data showing that the activated CD45RA^−^ FOXP3^hi^ Tregs increase with time from the relapse (45) and that patients with RRMS with lower percentages of naïve Tregs are the ones with highest expanded disability status scale (EDSS), an alternative scale of disability to MSSS (47).

The CD56^bright^ cells belong to the most immature NK cell subset and are described to be more potent at producing cytokines (48). We observed that patients with RRMS had a higher percentage of these cells expressing the inhibitory receptor KLRG1. KLRG1 binds E-, N-, and R-cadherins, being the last two found in the CNS. Specifically, N-cadherin was found to be increased in remyelinating lesions of EAE mice, particularly in oligodendrocytes (49). The increase in the percentage of KLRG1^+^ among CD56^bright^ NK cells might suggest that the most immature NK subset might be less prone to kill target cells in patients than the ones from healthy individuals, although functional studies should clarify this issue. Regarding the activating receptors, we observed that patients present a higher percentage of CD56^dim^CD57^+^ NK cells, the most mature and cytotoxic NK cell subset, expressing NKp30. Engagement of the natural cytotoxicity receptors NKp30, NKp44, and/or NKp46 from CD56^bright^ NK cells is required to suppress autologous T cells from untreated patients with RRMS *in vitro* (23). However, no significant suppression of autologous T cells from untreated patients with RRMS was observed by the most mature CD56^dim^ NK cells (23). Although, we cannot exclude a possible role of CD56^dim^CD57^+^ NK cells at controlling autoreactive T cells as a higher number of this cell subset was related with increasing time from the relapse.

Regarding NKT cells, despite patients with RRMS having a lower percentage of these cells expressing KIR3DL1, this difference was lost and mostly attributed to anti-HCMV IgG seroprevalence. Still, higher numbers of total NKT cells were related with higher time from the relapse, and higher percentages of these cells expressing the inhibitory receptors KIR2DL2/3 and KIR3DL1 are related to higher MS severity, an observation not previously described to our knowledge.

In summary, we observed profound peripheral immune alterations in newly diagnosed patients with RRMS including unbalanced naïve and memory cells due to deficiency on the memory subset, particularly on CD8^+^ T cells. Furthermore, these patients were characterized by alterations on regulatory cells, presenting more naïve Tregs, higher percentages of immature NK cells expressing inhibitory receptors and higher percentages of the most mature NK cells expressing activating receptors. Together, an unbalance on conventional T cells together with unbalanced regulatory cells subsets seems to characterize patients with RRMS at clinical diagnosis. Although MS initiates much earlier than symptoms onset, the characterization of the peripheral immune system cells in newly diagnosed patients with RRMS unveiled immune features present at disease clinical onset that might set the basis to the better understanding of the disease pathogenesis and the search for novel diagnostic biomarkers.

## Material and Methods

### Study Population

Patients included in this cross-sectional study were followed-up between 2018 and 2021 as outpatients at *Hospital de Braga* (Braga, Portugal)*, Hospital Geral de Santo António* (*Centro Hospitalar Universitário do Porto*, Porto, Portugal) and *Hospital Álvaro Cunqueiro* (Vigo, Spain). Patients older than 18 years old, newly diagnosed with RRMS according to McDonald 2017 criteria and naïve for MS disease modifying drugs, were recruited. Exclusion criteria: other immune disorders (other autoimmune or immunodeficiency diseases); history of radiotherapy and/or chemotherapy; on immunosuppressive drugs; treated with corticosteroids on the last 3 months or continuously for more than 6 months; splenectomy or thymectomy; and/or pregnancy. Data on age, sex, age of MS onset, months living with MS, months from the relapse, and disease severity [MS severity score (MSSS)] were retrieved from medical records (**eTable 12**). Healthy controls, age- and sex-matched, were recruited at *Centro Clínico Académico*, *Hospital de Braga*.

### Sample Processing

Blood was collected into K_3_EDTA tubes. Processing of blood samples from the different hospitals was centralized at the Life and Health Sciences Research Institute (ICVS; Braga, Portugal) and performed, in the exact same conditions for all samples, within 6 h after collection.

T cells and CD4^+^ T cells absolute numbers were determined in 10 μl of whole blood through: i) Muse Human CD4 T cell Kit (Millipore Corporation, CA, USA), according to manufacturer’s protocol, and acquired on a MUSE cell analyzer (Millipore Corporation); or ii) FACS staining with titrated anti-human PE-conjugated anti-CD3 (clone OKT3) and APC-Cy7-conjugated anti-CD4 (clone RPA-T4; both from BioLegend, CA, USA) for 15 min, followed by a 15 min incubation with red blood cells lysis buffer (Ammonium-Chloride-Potassium); prior to acquisition on a BD LSRII cytometer; a known number of counting beads (Molecular Probes, OR, USA) were added to each tube.

Peripheral blood mononuclear cells (PBMCs) and plasma were obtained from whole blood by Histopaque-1077 (Sigma-Aldrich, MO, USA) gradient centrifugation and enumerated. Plasma was aliquoted and frozen at −80°C. One million PBMCs were centrifuged at 16,540*g* and frozen as a dry pellet for quantification of T cell receptor excision circles (TRECs). The remaining PBMCs were frozen, in 5 million cells aliquots, in RPMI media supplemented with 20% fetal bovine serum (FBS) (Pan Biotech, Aidenbach, Germany) and 10% dimethyl sulfoxide (DMSO; Sigma-Aldrich, MO, USA) and kept at −80°C within a MrFrosty containing isopropanol for at least 24 h prior being transferred to liquid nitrogen.

### Anti-Human Cytomegalovirus IgG Seroprevalence

Semi-quantitative measurement of plasma IgG antibodies against the human cytomegalovirus (HCMV) was performed using the anti-HCMV IgG Elisa kit (Abcam, Cambridge, UK), according to manufacturer’s instructions.

### FACS Staining

PBMCs aliquots were thawed in a water bath and washed using FACS buffer (PBS with 2% BSA and 0.01% sodium azide). One million PBMCs per stain were incubated with the following antibody panels (**eTable 13**): i) Recent Thymic Emigrants (RTEs) homeostasis; ii) CD4^+^ and CD8^+^ T cells subsets homeostasis; iii) Tregs characterization; and iv) inhibitory and activating NK/NKT cells study. After incubation for 20 min, room temperature (RT) in the dark, cells were thoroughly washed with FACS buffer or PBS. The RTEs homeostasis panel was acquired on the same day after incubation with 7-AAD for 10 min. Cells from the remaining panels were incubated with a fixable viability dye (30 min, RT, dark), and fixed using the Fixation/Permeabilization solution from the FOXP3 Staining Buffer Set (eBiosciences, CA, USA). Cells from the Tregs stain were further permeabilized using the permeabilization buffer from the same kit followed by intracellular staining of FOXP3 (30 min, RT, dark). For the CD4^+^ and CD8^+^ T cells subsets homeostasis and Tregs characterization panels, all samples were analyzed in a single batch. As the samples for the RTE and NK/NKT cells panel were analyzed in several batches, a reference control consisting of PBMCs from one individual was always stained, acquired, and analyzed together with study samples to control for experimental and analysis variability. The Coefficient of Variation (CV) of the reference control sample was extracted for each cell population; the mean CV of all populations was 7.4%, with the lowest CV of 0.1% for the percentage of KLRG1^+^ NK cells, and the highest CV of 18.9% for the percentage of NKp30^+^ NK cells. Moreover, prior to acquisitions, rainbow calibration beads (BioLegend, CA, USA) were acquired, and voltages adjusted to maintain cytometer settings standardization between different experimental batches.

Samples were acquired on a BD LSRII flow cytometer, using the FACS Diva Software v6.0 (Becton Dickinson, NJ, USA). Data were analyzed in a blinded way, using the FlowJo Software v10 (Becton Dickinson, NJ, USA), as described in **Supplementary eFigures 4**–**7**. Tregs subsets and inhibitory and activating receptors’ expression among NK and NKT cells were evaluated only when the parent population had at least 500 events.

### T Cell Receptor Excision Circles Quantification

Single-joint (sj) and Dβ-Jβ (DJβ) TRECs were quantified through nested polymerase chain reaction (PCR), using primers and standard curve’s plasmids, as previously described by Dion et al. and adapted by us as follows (28). Dry pellets of 1 million PBMCs were thawed (10 min, RT) resuspended in 0.1 ml of lysis solution [10 mM Tris-HCl pH 8.0, Tween-20 (0.05%), 0.05% nonidet P-40 (NP-40 from Calbiochem; Merck KGaA, Darmstadt, Germany), and 100 μg/ml Proteinase K (Grisp, Porto, Portugal)], and incubated on a thermocycler (Mastercycler epgradient S; Eppendorf, Hamburg, Germany) for 30 min at 56°C, followed by 10 min at 98°C. Samples were kept at 4°C until being tested. sjTRECs and DJβTRECs were quantified in distinct assays, each together with CD3; two plasmids were used for quantification, each containing the sjTRECs or DJβTRECs sequence and the housekeeping gene CD3 at a 1:1 ratio (28). The first round of amplification was performed on cell lysates and plasmid through a multiplex PCR using specific 5′/3′ “out” primers to sjTRECs/CD3 or DJβTRECs/CD3 (**eTable 14**) and 0.5 U Xpert Taq DNA Polymerase, 2.5 mM MgCl_2_ and 0.4 mM dNTPs mix (all from Grisp): 15 min at 95°C followed by 19 amplification cycles of 30 s at 95°C, 30 s at 60°C and 3 min at 72°C. The sample products of the first amplification were 10- or 50-fold diluted for sjTRECs/CD3 or DJβTRECs/CD3, respectively; the standard curve was prepared through eight sequential 10-fold dilutions of the plasmid first amplification product. Using specific 5′/3′ “in” primers (**eTable 14**) and 1× Kapa SYBR Fast qPCR Master Mix (Roche, Basel, Switzerland), TRECs and CD3 were amplified in independent wells, each in duplicate in the same qPCR run using either a Bio-Rad CFX96 real-time system with a C1000 thermal cycler (Bio-Rad, CA, USA) or a Applied Biosystems 7500 Real-Time PCR Systems (Thermo Fisher Scientific, MA, USA): 15 min at 95°C for, followed by 40 cycles of 2 s at 95°C, 15 s at 62°C or 60°C for sjTREC/CD3 or DJβTRECs/CD3, respectively, and 10 s at 72°C. The fluorescence signal was measured in each well at the end of each cycle. Melting curves were analyzed and showed a single sharp peak at the temperature characteristic of the primers used. Data were analyzed in Bio-Rad CFX Manager v3.1 (Bio-Rad) or Design and Analysis Software v2.4.3 (Thermo Fisher Scientific) and sjTREC and DJβTRECs quantification represented as number of TRECs/10^5^ PBMCs. The sj/DJβTREC ratio was calculated as previously described (28).

### Statistical Analysis

Differences were considered statistically significant for *p*-values <0.050. The histogram and measures of asymmetry and kurtosis were evaluated, and the D’Agostino and Pearson tests performed to assess the normality assumption for parametric tests; for data not following a normal distribution, non-parametric tests were used. For quantitative data, depending on the underlying distributions, comparisons between two independent groups were performed through independent *t*-test or Mann–Whitney *U*-test. For qualitative data, comparison between two groups was performed through the Chi-square test. As a measure of the magnitude of a difference, the effect size (practical significance) was calculated as follows (50):

- *t*-tests, as the groups have unequal sample sizes, Cohen’s *d* was calculated as follows:

$$d=\frac{M_{1}-\;M_{2}}{\sqrt{\frac{(N_{1}-1)SD_{1}^{2}+(N_{2}-1)SD_{2}^{2}}{\left(N_{1}-\;N_{2}\right)}}}$$

where

- M_1_ and M_2_: Mean of group 1 and group 2, respectively.
- N_1_ and N_2_: Sample size of group 1 and group 2, respectively.
- SD_1_ and SD_2_: Standard deviation of group 1 and group 2, respectively.

Cohen’s *d* was considered small if *d <*0.300; medium if *d* = [0.300,0.800]; or large if *d >*0.800.

- Mann–Whitney *U*-test, *r* was calculated as follows:

$$r=\frac{Z}{\sqrt{N}}$$

where

- Z: z-score.
- N: Total sample size.

r was considered small if *r <*0.300, medium if *r* = [0.300,0.500], or large if *r >*0.500. Sample size, statistical outputs, and effect size are summarized in **eTable 1**. As sex, age, and anti-HCMV IgG seroprevalence contribute to variations on immune cells percentages and numbers (51, 52), all comparisons were confirmed through multiple linear regression models to adjust for these variables. The dependent variable was the cell population in study (either frequency or number), and the independent variables age, sex (reference category: female), anti-HCMV IgG seroprevalence (reference: IgG negative), and RRMS (reference: healthy). All differences between study groups were maintained in those models (**eTables 2–9**), unless otherwise stated in the results section. MS clinical parameters contribution to immune cell populations alterations were evaluated through sequential multiple linear regressions followed by simple linear regressions. First, to control for age, sex, and anti-HCMV IgG seroprevalence multiple linear regressions having as dependent variable each of the cell populations were performed, and the unstandardized residuals retrieved (53). Then, the predictive value of each MS clinical parameter was evaluated through simple linear regression having as dependent variable the abovementioned unstandardized residuals for each cell population, and as independent variables either the time from the relapse or the MS severity score (**eTables 10, 11**). Corticosteroids’ administration was included also as an independent dichotomous variable in the regression for time from the relapse to control for the possible residual consequences of corticosteroids administration for relapse treatment. Effect size *R* ^2^ for the linear regression was considered small, medium, and large if *R* ^2^ <0.090, *R* ^2^ = [0.090; 0.250], and *R* ^2^ >0.250, respectively.

The estimated sample size was inferred based on the minimum sample size required to ensure an accurate prediction. According to Tabachnick and Fidell (1989), five individuals are the minimum required sample size to include in a multiple linear regression model per predictor (54). In our study, the multiple linear regression models included: i) four predictors (**eTables 2–9**) for a sample size that ranged from 31 to 63 individuals (depending on the cell population); ii) two predictors (**eTable 10**) for a sample size that ranged from 26 to 28 individuals, and; iii) one predictor (**eTable 11**) for a sample size that ranged from 20 to 29 individuals.

Statistical analyses were performed using IBM SPSS Statistics v26 (IBM Corporation, NY, USA) and GraphPad Prism v9 (GraphPad Software, CA, USA).

## Data Availability Statement

The original contributions presented in the study are included in the article/**Supplementary Material**. Further inquiries can be directed to the corresponding author.

## Ethics Statement

The studies involving human participants were reviewed and approved by Hospital de Braga (ref. 5888/2016-CESHB), Centro Hospitalar Universitário do Porto (ref. 098-DEFI/097-CES), and Hospital Álvaro Cunqueiro (ref. 2021/430). The patients/participants provided their written informed consent to participate in this study.

## Author Contributions

JC-G, MC-N, JJC, and CN contributed to conception and design of the study. JC-G, CS, RR-S, and CN performed the experimental tasks. JJC, AMdS, DB, and IG-S included patients and provided clinical data. RC provided key resources. JC-G organized the database. JC-G, CN, and PC performed the statistical analysis. JC-G and CN generated the figures and tables. JC-G and CN wrote the first draft and all authors contributed to manuscript revision. All authors contributed to the article and approved the submitted version.

## Funding

The work presented was performed at Life and Health Sciences Research Institute (ICVS), University of Minho. This work has been funded by a research grant from the Academic Clinical Centre of Hospital of Braga and by National funds, through the Foundation for Science and Technology (FCT) - project UIDB/50026/2020 and UIDP/50026/2020 and by the project NORTE-01-0145-FEDER-000039, supported by Norte Portugal Regional Operational Programme (NORTE 2020), under the PORTUGAL 2020 Partnership Agreement, through the European Regional Development Fund (ERDF). JC-G is supported by an FCT PhD grant, in the context of the Doctoral Program in Aging and Chronic Diseases (PD/BD/137433/2018). CS is supported by an FCT PhD grant, in the context of the Doctoral Program in Applied Health Sciences (PD/BDE/142976/2018). CN is a junior researcher under the scope of the FCT Transitional Rule DL57/2016. The funders had no role in study design, data collection and analysis, decision to publish, or preparation of the manuscript.

## Conflict of Interest

The authors declare that the research was conducted in the absence of any commercial or financial relationships that could be construed as a potential conflict of interest.

## Publisher’s Note

All claims expressed in this article are solely those of the authors and do not necessarily represent those of their affiliated organizations, or those of the publisher, the editors and the reviewers. Any product that may be evaluated in this article, or claim that may be made by its manufacturer, is not guaranteed or endorsed by the publisher.

## Supplementary Material

Supplementary eFigure 1 Newly diagnosed patients with RRMS present no differences on T helper (Th) subsets. Percentages and numbers of Th subsets are represented for healthy controls (white circles) and newly diagnosed RRMS patients (black circles). Percentages and numbers of Th cell subsets: Th1 **(A)**, Th2 **(B)**, Th9 **(C)**, Th17 **(D)**, Th22 **(E)**, and Th-GM-CSF (ThG; **F**). The parametric Student’s *t-*test was performed in (B, D; percentage); the non-parametric Mann–Whitney *U*-test in **(A)**, (**B**; number), **(C)**, (**D**; number), and **(E**, **F)** (statistical outputs and effect size calculations in **eTable 1**). In all graphs, each dot represents one individual, and the horizontal lines the groups’ means or medians depending on the normal or non-normal distribution of the data, respectively. Results are maintained upon controlling for sex, age, and human cytomegalovirus IgG seroprevalence on multiple linear regression models (**eTable 4**), except for the Th2 cells **(B)** whose number becomes tendentially lower in patients.

Supplementary eFigure 2 Newly diagnosed patients with RRMS present lower memory CD8^+^ T cells expressing the chemokine receptor CCR6. Percentages and numbers of chemokine receptors expressing CD8^+^ T cells subsets are represented for healthy controls (white circles) and newly diagnosed RRMS patients (black circles). Percentages and numbers of CCR4, CCR6, CCR10, and CxCR3 are represented among: total memory **(A)**, central memory **(B)**, effector memory **(C)**, and TemRA **(D)**. The parametric Student’s *t-*test was performed in **(B**; CCR6 and CxCR3 percentages) and **(D**; CCR6 percentage); the non-parametric Mann–Whitney *U*-test in **(A)**, (**B**; CCR6 and CxCR3 number and in CCR4 and CCR10), **(C)**, (**D**; CCR6 number and in CCR4, CCR10, and CxCR3); statistical outputs and effect size calculations in **eTable 1**. In all graphs, each dot represents one individual, and the horizontal lines the groups’ means or medians depending on the normal or non-normal distribution of the data, respectively. When differences were statistically significant (*p-value <*0.05), the *p*-value was represented by * for 0.010< p <0.050; ** 0.001< *p ≤*0.010; and **** *p≤*0.0001. Results are maintained upon controlling for sex, age, and human cytomegalovirus (HCMV) IgG seroprevalence on multiple linear regression models (**eTable 6**), except for the central memory CD8^+^ T cells expressing CCR6 **(A)**, where a statistical tendency to lower number of those cells became evident in patients and for the effector memory CD8^+^ T cells expressing CCR6 **(C)**, whose lower number observed in patients was lost due to sex and HCMV IgG seroprevalence.

Supplementary eFigure 3 Newly diagnosed patients with RRMS have lower percentages of NKT cells expressing the inhibitory receptor KIR3DL1. Percentages and numbers of natural killer (NK) T cells are represented for healthy controls (white circles) and newly diagnosed RRMS patients (black circles; **A)**. Percentage of cells expressing inhibitory receptors in NKT cells: KLRG1 **(B)**, NKG2A **(C)**, KIR2DL2/3 **(D)**, and KIR3DL1 **(E)**. Percentage of cells expressing activating receptors in NKT cells: NKp30 **(F)**, NKp44 **(G)** and NKp46 **(H)**. In all graphs, each dot represents one individual, and the horizontal lines the groups’ medians, as data follow a non-normal distribution. The non-parametric Mann–Whitney *U*-test was performed to compare groups (statistical outputs and effect size calculations in **eTable 1**). When differences were statistically significant (*p-value <*0.050), the *p*-value was represented by * for 0.010< p <0.050. Results are maintained upon controlling for sex, age, and human cytomegalovirus (HCMV) IgG seroprevalence on multiple linear regression models (**eTable 9**), except for the percentage of KIR3DL1^+^ NKT cells where the statistical significant difference is lost due to the HCMV IgG seroprevalence **(E)**.

Supplementary eFigure 4 Gating strategy for phenotypical analysis of recent thymic emigrants. Issues during acquisition were verified by accompanying acquisition throughout time **(A)**. Leukocytes were then selected by their positive expression of CD45 **(B)**, and viable cells selected by excluding 7-AAD positive cells **(C)**. After isolating the singlets **(D)**, the lymphocytes were gated regarding their lower size (FSC-A) and complexity (SSC-A; **E)**. Within the lymphocytes population T cells were defined through the expression of CD3^+^ **(F)**. CD4^+^ were then discriminated among T cells **(G)**. CD45RA and CCR7 markers were used to discriminate the Naïve subset (CD45RA^+^ exclude CCR7^+^; **H)**. Finally, recent thymic emigrants were defined by the expression of the CD31 marker among the naïve CD4^+^ T cell subset (CD45RA^+^CCR7^+^CD31^+^; **I)**.

Supplementary eFigure 5 Gating strategy for phenotypical analysis of the main T cell subsets. Issues during acquisition were verified and excluded using the FlowAI plugin in FlowJo. Singlets were isolated **(A)** and live leukocytes selected by their positive expression of CD45 and exclusion of the most positive cells for the viability dye **(B)**. The lymphocytes were gated regarding their lower size (FSC-A) and complexity (SSC-A; **C)**. Within the lymphocyte population, T cells were defined by the positive expression of CD3 **(D)**. CD4^+^ and CD8^+^ cell subsets were then discriminated among T cells **(E)**. CD45RA and CCR7 were used to distinguish both for CD4^+^ **(H)** and CD8^+^ **(I)** T cells the Naïve (CD45RA^+^CCR7^+^), Central Memory (CD45RA^-^CCR7^+^), Effector Memory (CD45RA^−^CCR7^−^) and terminally differentiated CD45RA-expressing memory T cells (TemRA; CD45RA^+^CCR7^-^) subsets **(F, G)**. Finally, T helper (Th) subsets were defined among total memory CD4^+^ T cells through the differential expression of CCR4, CCR6, CCR10 and CxCR3 **(H–K)**: Th1 (CxCR3^+^); Th2 (CCR4^+^CCR6^−^CCR10^−^CxCR3^−^); Th9 (CCR6^+^CCR4^−^); Th17 (CCR4^+^CCR6^+^CCR10^−^CxCR3^−^); Th22 (CCR4^+^CCR6^+^CCR10^+^CxCR3^−^) and ThG (CCR4^+^CCR6^-^CCR10^+^CxCR3^−^).

Supplementary eFigure 6 Gating strategy for phenotypical analysis of regulatory T cells (Tregs). Issues during acquisition were verified and excluded using the FlowAI plugin in FlowJo. Singlets were selected **(A)** and live leukocytes discriminated by their positive expression of CD45 and by excluding the most positive cells for the viability dye **(B)**. The lymphocytes were gated according to their lower size (FSC-A) and complexity (SSC-A; **C)**. Within the lymphocytes, T cells were defined by the expression of CD3^+^ **(D)** followed by discrimination of the CD4^+^ and CD8^+^ T cell subsets **(E)**. Tregs were defined after selection of cells expressing low to intermediate levels of CD127 **(F)** followed by selection of FoxP3 and CD25 positive CD4^+^ T cells **(G)**. The CD45RA and HLA-DR markers were used to define three Tregs subsets: the naïve (CD45RA^+^HLA-DR^−^), the activated HLA-DR^−^ (CD45RA^−^HLA-DR^−^) and the activated HLA-DR^+^ (CD45RA^−^HLA-DR^+^) Tregs **(H)**. Suppressive markers were also evaluated among total Tregs: CD39 **(I)**, CD73 **(J)**, and GARP **(K)**.

Supplementary eFigure 7 Gating strategy for phenotypical analysis of NKT and NK subsets regarding expression of inhibitory and activating receptors. Issues during acquisition were verified by accompanying acquisition throughout time **(A)**. Live leukocytes were then selected by their positive expression of CD45 and by excluding the most positive cells for the viability dye **(B)**. After isolating the singlets **(C)**, the lymphocytes were gated regarding their size (FSC-A) and complexity (SSC-A; **D)**. Within the lymphocytes population NK and NKT cells were defined by their positive expression of CD56 and differential expression of CD3 **(E)**. NK cell subsets were then discriminated according to the expression of CD57 and level of expression of CD56 into three main populations: CD56^bright^ NK cells, CD56^dim^CD57^-^ NK cells and CD56^dim^CD57^+^ NK cells. Depending on the staining panel the expression of inhibitory (KIR2DL2/3, KIR3DL1, KLRG1, and NKG2A) or of activating receptors (NKp30, NKp44, and NKp46) were evaluated within the NK subsets and NKT cells **(G)**.
