# Supplementary figures and images for: Low Memory T Cells Blood Counts and High Naïve Regulatory T Cells Percentage at Relapsing Remitting Multiple Sclerosis Diagnosis
Source: Front Immunol. 2022 May 30;13:901165. doi: 10.3389/fimmu.2022.901165 (PMC9196633; doi:10.3389/fimmu.2022.901165)

# eFigure 1

A

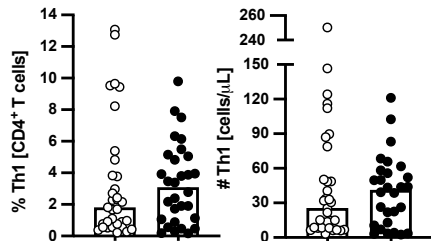

B

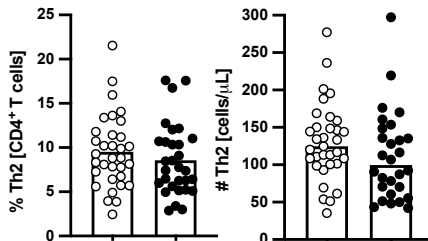

C

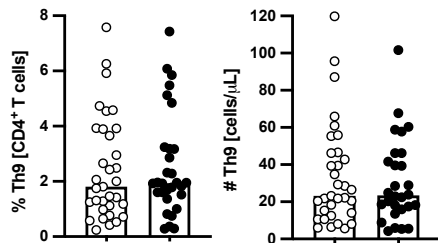

D

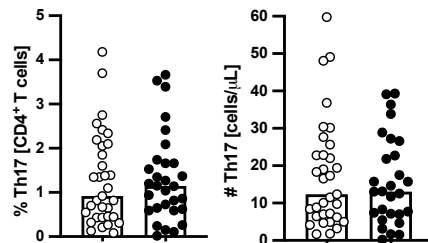

E

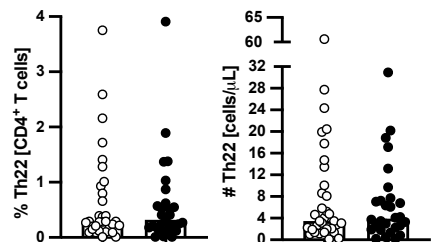

F

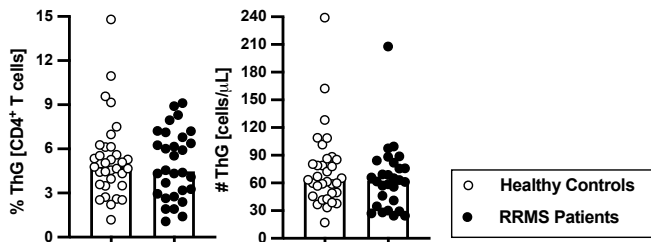

eFigure 2

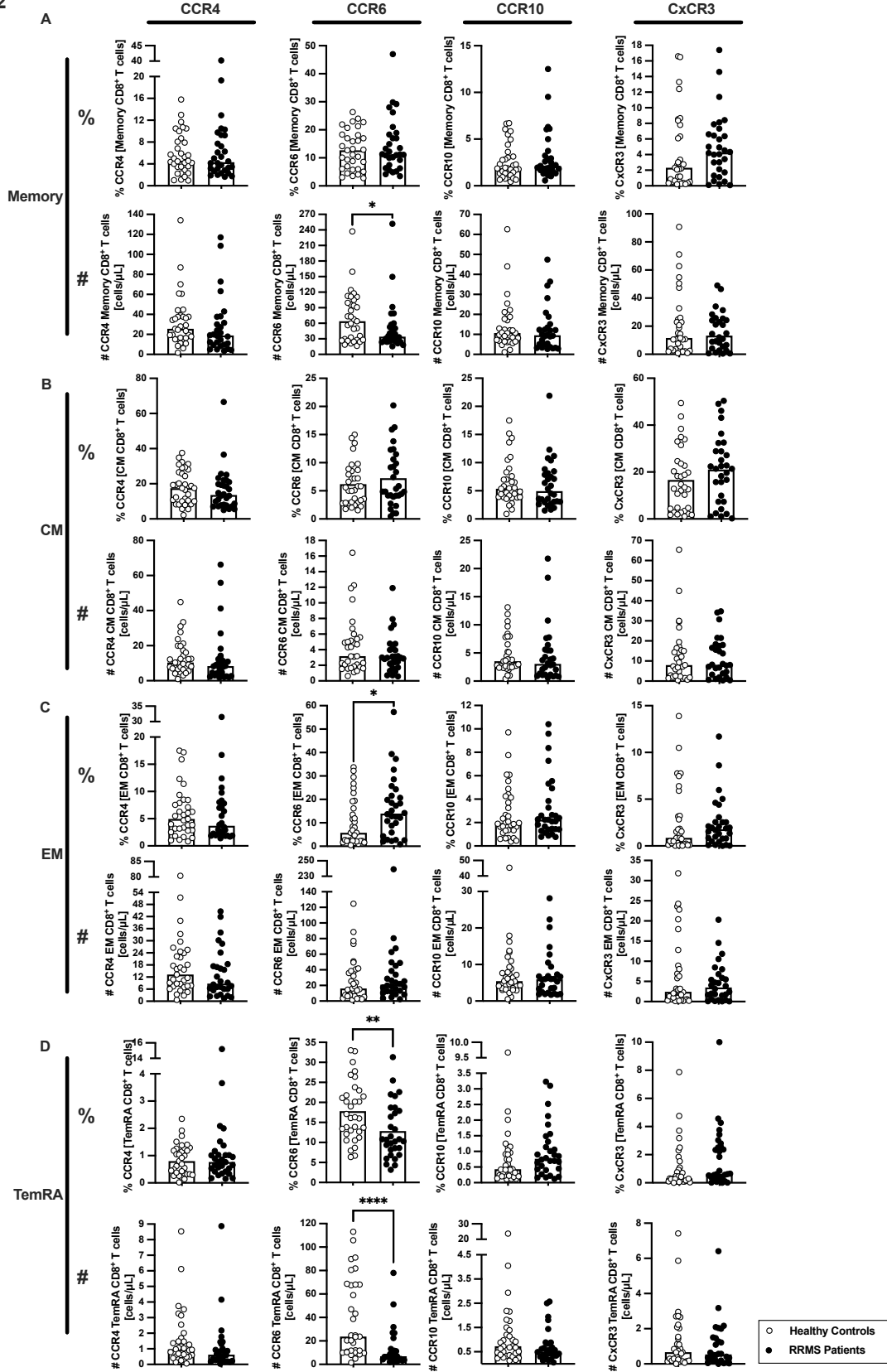

# eFigure 3

**A**

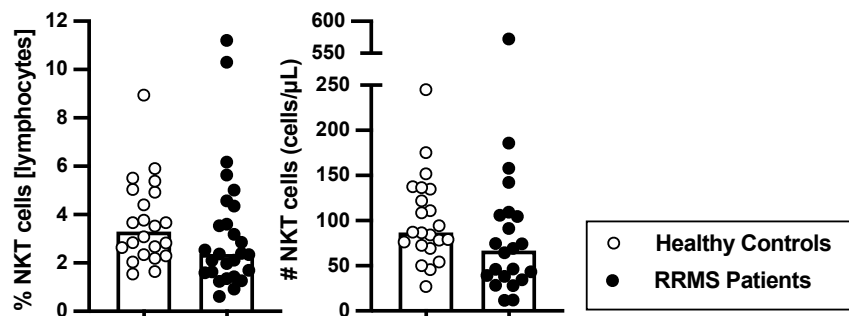

**B**

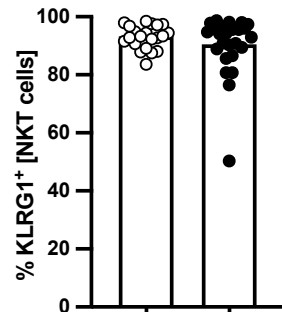

**C**

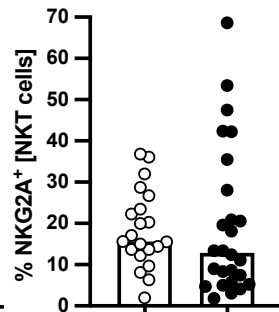

**D**

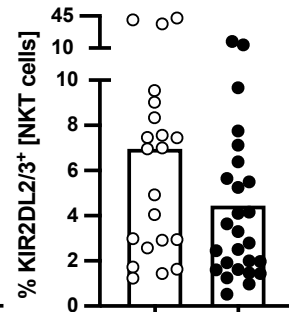

**E**

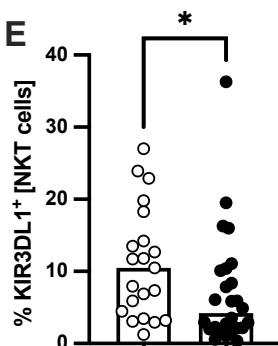

**F**

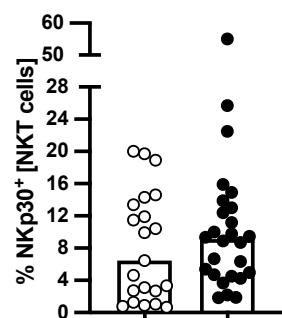

**G**

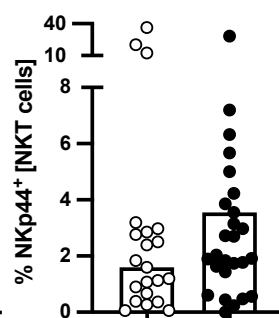

**H**

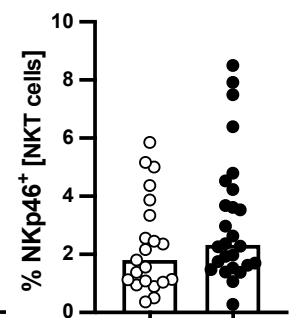

eFigure 4

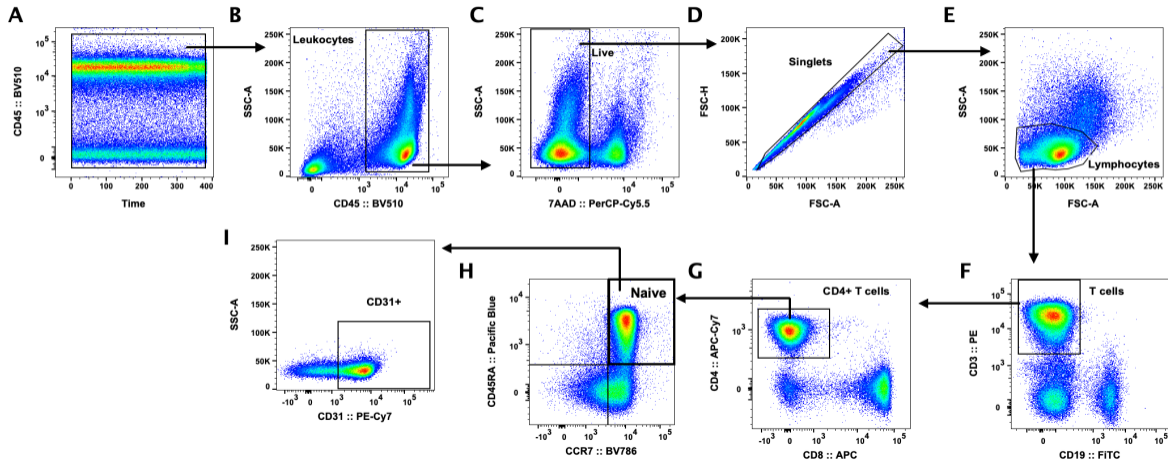

**eFigure 5**

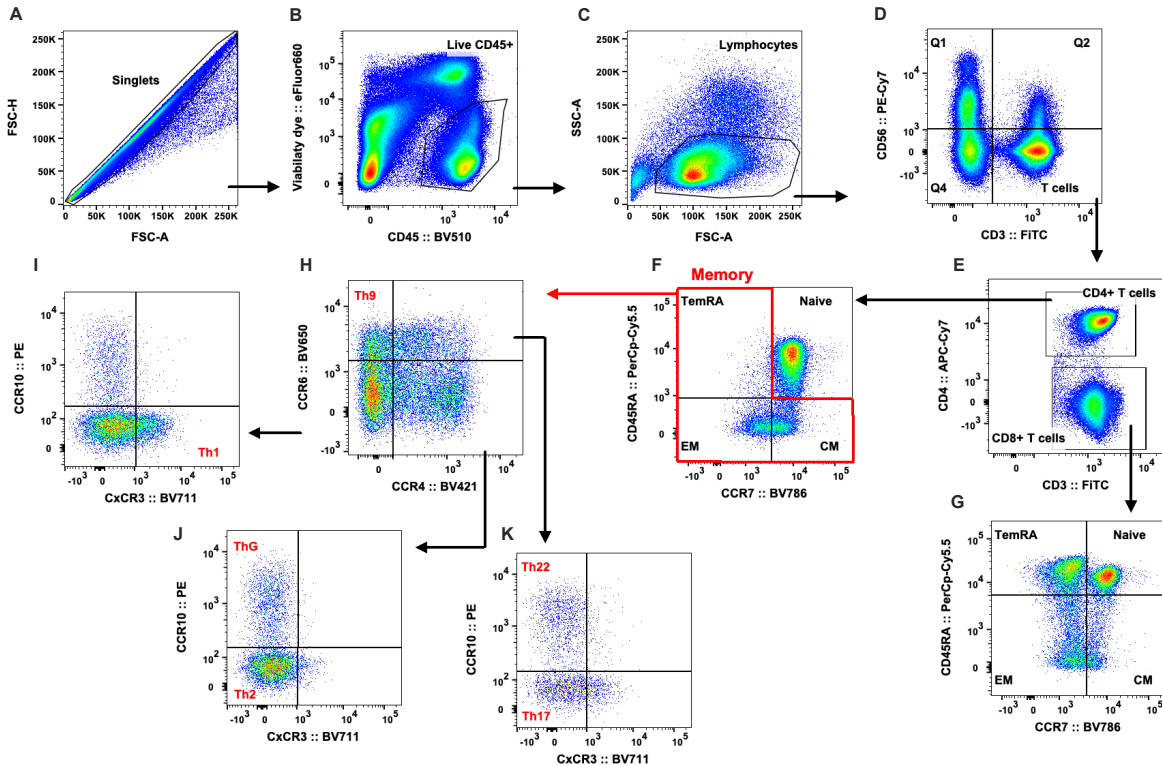

eFigure 6

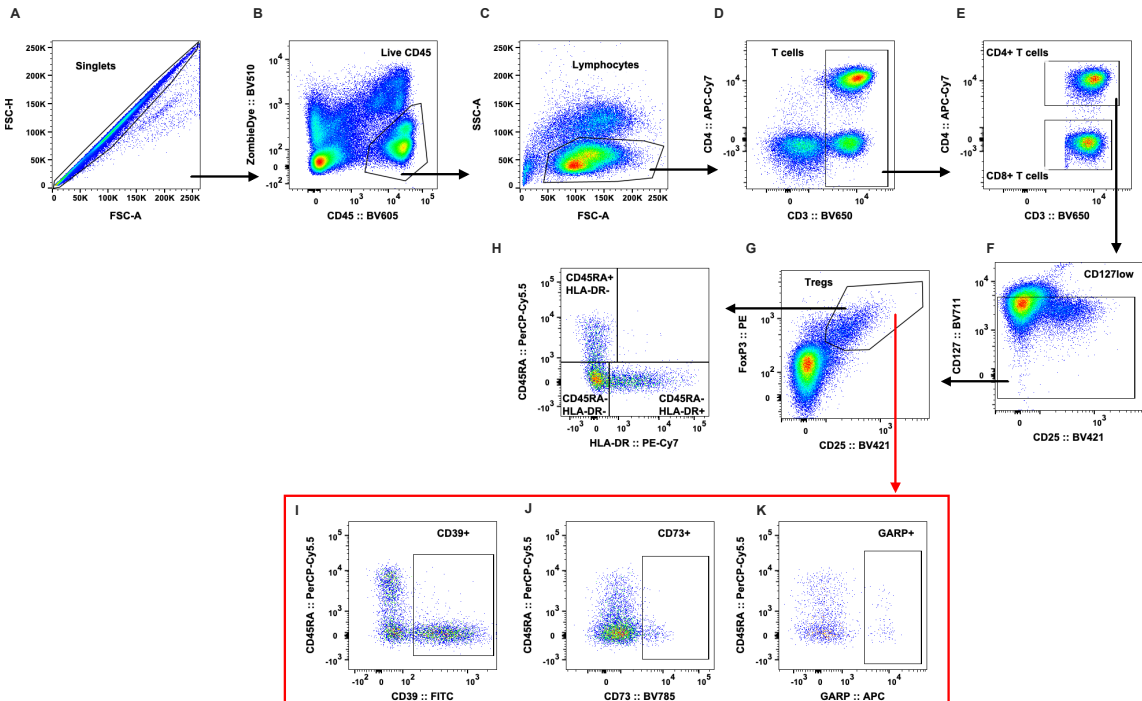

eFigure 7

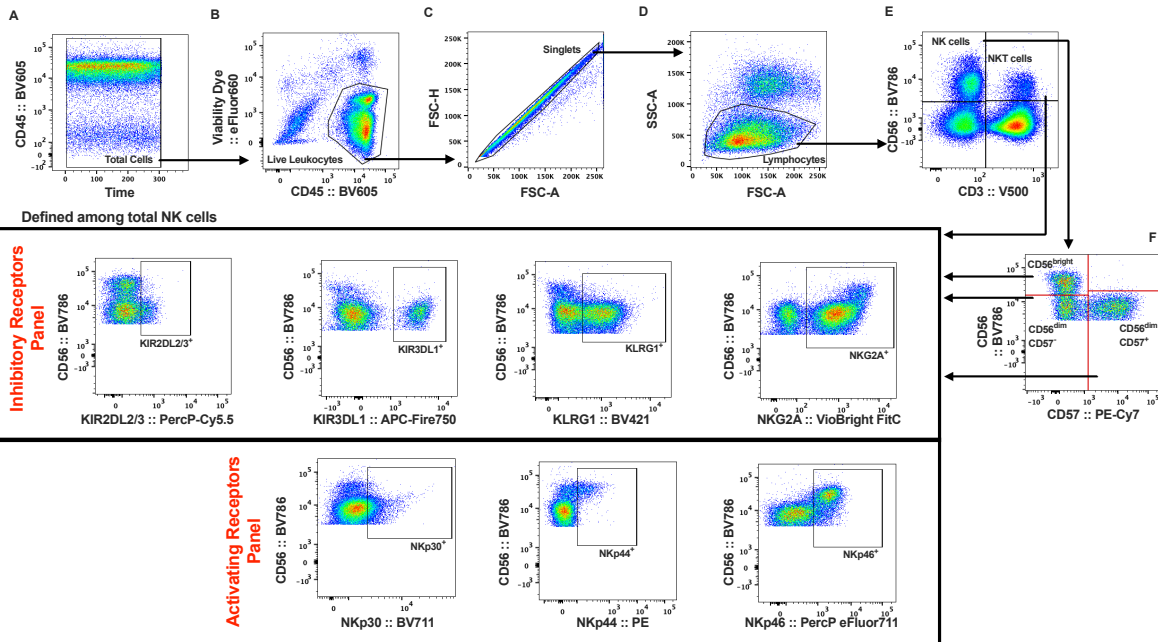

Supplement: Supplementary eFigure 1 — Newly diagnosed patients with RRMS present no differences on T helper (Th) subsets. Percentages and numbers of Th subsets are represented for healthy controls (white circles) and newly diagnosed RRMS patients (black circles). Percentages and numbers of Th cell subsets: Th1 (A), Th2 (B), Th9 (C), Th17 (D), Th22 (E), and Th-GM-CSF (ThG; F). The parametric Student’s t-test was performed in (B, D; percentage); the non-parametric Mann–Whitney U-test in (A), (B; number), (C), (D; number), and (E, F) (statistical outputs and effect size calculations in eTable 1 ). In all graphs, each dot represents one individual, and the horizontal lines the groups’ means or medians depending on the normal or non-normal distribution of the data, respectively. Results are maintained upon controlling for sex, age, and human cytomegalovirus IgG seroprevalence on multiple linear regression models ( eTable 4 ), except for the Th2 cells (B) whose number becomes tendentially lower in patients. [file DataSheet_1.pdf]
