# Supplementary material for: Low Memory T Cells Blood Counts and High Naïve Regulatory T Cells Percentage at Relapsing Remitting Multiple Sclerosis Diagnosis
Source: Front Immunol. 2022 May 30;13:901165. doi: 10.3389/fimmu.2022.901165 (PMC9196633; doi:10.3389/fimmu.2022.901165)
Supplement: Supplementary file 2 [file Table_1.pdf]

eTable 1. Statistical outputs and sample size calculations.

	Percentage of cells					Cells absolute numbers					Fig.
	Sample Size		Test result	*p-value*[1]	Effect size[2]	Sample Size		Test result	*p-value*[1]	Effect size[2]	
	HC	RRMS				HC	RRMS				
**CD31$^+$ naive CD4$^+$ T cells**	20	19	U = 137,5	0,143	0,223	20	19	U = 163	0,628	0,065	1A
**sj/DJβTREC**	16	16	t_{30} = 0,157	0,876	0,092						1B
**sjTRECs**	20	19	U = 139,0	0,155	0,225						1C
**DJβTREC**	16	16	U = 98,000	0,179	0,226						1D
**CD4$^+$ T cells**	33	30	t_{61} = 1,722	0,090	0,434	33	28	t_{59} = 1,413	0,163	0,361	2A
**Naive/Memory CD4$^+$ T cells**	33	30	U = 413,0	0,263	0,141						2C
**Naïve CD4$^+$ T cells**	33	30	t_{61} = 0,969	0,336	0,244	33	28	U = 426,0	0,607	0,067	2D
**Memory CD4$^+$ T cells**	33	30	t_{61} = 0,969	0,336	0,244	33	28	U = 356,0	0,127	0,196	2E
**CM CD4$^+$ T cells**	33	30	t_{61} = 0,575	0,568	0,145	33	28	U = 392,5	0,319	0,130	2F
**EM CD4$^+$ T cells**	33	30	U = 391	0,154	0,180	33	28	U = 351,500	0,111	0,204	2G
**TemRA CD4$^+$ T cells**	33	30	U = 368,0	0,081	0,220	33	28	U = 337,5	0,072	0,228	2H
**CD8$^+$ T cells**	33	30	U = 365,500	0,075	0,225	33	28	t_{59} = 2,647	0,010	0,682	3A
**Naive/Memory CD8$^+$ T cells**	33	30	U = 366,0	0,076	0,225						3C
**Naïve CD8$^+$ T cells**	33	30	t_{61} = 2,151	0,035	0,543	33	28	t_{59} = 0,664	0,509	0,171	3D
**Memory CD8$^+$ T cells**	33	30	t_{61} = 2,151	0,035	0,543	33	28	t_{59} = 3,325	0,002	0,859	3E
**CM CD8$^+$ T cells**	33	30	t_{61} = 0,925	0,358	0,233	33	28	U = 364,5	0,160	0,176	3F
**EM CD8$^+$ T cells**	33	30	U = 492	0,970	0,005	33	28	U = 311,000	0,028	0,280	3G
**TemRA CD8$^+$ T cells**	33	30	U = 298,0	0,006	0,342	33	28	U = 230,5	0,001	0,428	3H
**Treg**	33	30	U = 432,5	0,394	0,108	33	28	U = 434,0	0,690	0,056	4A
**Naive/Memory Tregs**	33	30	U = 316,5	0,013	0,310						4C
**CD45RA$^+$HLA-DR$^-$ Tregs**	33	30	U = 317	0,014	0,309	33	28	t_{59} = 1,45	0,152	0,371	4D
**CD45RA$^-$HLA-DR$^-$ Tregs**	33	30	t_{61} = 1,888	0,064	0,476	33	28	U = 367,0	0,171	0,178	4E
**CD45RA$^-$HLA-DR$^+$ Tregs**	33	30	U = 381,5	0,120	0,197	33	28	U = 380,5	0,241	0,157	4F
**CD39$^+$ Tregs**	33	30	t_{61} = 2,019	0,048	0,509	33	28	U = 354,0	0,120	0,200	4G
**CD73$^+$ Tregs**	33	30	U = 464,000	0,674	0,054	33	28	U = 416,0	0,511	0,085	4H
**GARP$^+$ Tregs**	33	30	U = 286,000	0,004	0,362	33	28	U = 289,0	0,012	0,321	4I
**NK cells**	22	27	U = 295,5	0,980	0,004	22	22	U = 215,0	0,538	0,096	5A
**CD56^{bright} NK cells**	22	27	U = 247,0	0,320	0,144	22	22	t_{42} = 1,156	0,254	0,349	5B
**CD56^{dim}CD57$^-$ NK cells**	22	27	t_{47} = 0,913	0,366	0,262	22	22	U = 237,0	0,917	0,018	5C
**CD56^{dim}CD57$^+$ NK cells**	22	27	t_{47} = 1,329	0,190	0,382	22	22	U = 215,0	0,538	0,096	5D
**KLRG1$^+$ CD56^{bright} NK cells**	17	22	U = 215,5	0,006	0,262						
**KLRG1$^+$ CD56^{dim}CD57$^-$ NK cells**	21	26	t_{45} = 0,040	0,969	0,012						5E
**KLRG1$^+$ CD56^{dim}CD57$^+$ NK cells**	21	26	t_{45} = 1,072	0,289	0,315						
**NKG2A$^+$ CD56^{bright} NK cells**	17	22	U = 185,5	0,056	0,359						
**NKG2A$^+$ CD56^{dim}CD57$^-$ NK cells**	21	26	t_{45} = 1,832	0,074	0,538						5F
**NKG2A$^+$ CD56^{dim}CD57$^+$ NK cells**	21	26	t_{45} = 1,994	0,052	0,585						
**KIR2DL2/3$^+$ CD56^{bright} NK cells**	17	22	t_{37} = 0,187	0,852	0,061						
**KIR2DL2/3$^+$ CD56^{dim}CD57$^-$ NK cells**	21	26	t_{45} = 0,427	0,672	0,125						5G
**KIR2DL2/3$^+$ CD56^{dim}CD57$^+$ NK cells**	21	26	U = 280,5	0,795	0,048						
**KIR3DL1$^+$ CD56^{bright} NK cells**	17	22	U = 293,0	0,732	0,013						
**KIR3DL1$^+$ CD56^{dim}CD57$^-$ NK cells**	21	26	U = 248,0	0,320	0,144						5H
**KIR3DL1$^+$ CD56^{dim}CD57$^+$ NK cells**	21	26	U = 238,0	0,235	0,173						
**NKp30$^+$ CD56^{bright} NK cells**	17	15	U = 243,0	0,143	0,192						
**NKp30$^+$ CD56^{dim}CD57$^-$ NK cells**	22	27	t_{47} = 0,135	0,893	0,038						5I
**NKp30$^+$ CD56^{dim}CD57$^+$ NK cells**	22	26	U = 162,0	0,009	0,392						
**NKp44$^+$ CD56^{bright} NK cells**	17	15	U = 193,5	0,395	0,368						
**NKp44$^+$ CD56^{dim}CD57$^-$ NK cells**	22	27	U = 234,0	0,209	0,181						5J
**NKp44$^+$ CD56^{dim}CD57$^+$ NK cells**	22	26	U = 278,0	0,626	0,055						
**NKp46$^+$ CD56^{bright} NK cells**	17	15	t_{30} = 0,294	0,771	0,109						
**NKp46$^+$ CD56^{dim}CD57$^-$ NK cells**	22	27	U = 247,0	0,320	0,144						5K
**NKp46$^+$ CD56^{dim}CD57$^+$ NK cells**	22	26	t_{46} = 1,035	0,306	0,301						
**Th1 CD4$^+$ T cells**	33	30	U = 439,500	0,450	0,095	33	28	U = 439,000	0,747	0,043	e1A
**Th2 CD4$^+$ T cells**	33	30	t_{61} = 0,9254	0,358	0,233	33	28	U = 349,0	0,104	0,209	e1B
**Th9 CD4$^+$ T cells**	33	30	U = 463	0,664	0,055	33	28	U = 443,0	0,790	0,035	e1C

[1] Red cells refere to significant differences (p-value <0.050) and grey cells to tendencies (0.050< p-value <0.010)

[2] Cohen's d was calculated for t-tests, and r for Mann-Witney U-tests. Green cells refere to large effect sizes (d>0.800; r>0.500) and yellow cells to medium effect sizes (0.300≤d≤0.800; 0.300≤r≤0.500)

CM, Central Memory; **EM**, Effector Memory; **NK**, Natural Killer; **NKT**, Natural Killer T cells; **TemRA**, Terminally differentiated CD45RA-expressing memory cells; **Th**, T helper cells; **TREC**, T cell receptor excision circles; **Treg**, Regulatory T cells.

eTable 1 (cont.)

	Percentage of cells					Cells absolute numbers					
	Sample Size		Test result	*p-value*[1]	Effect size[2]	Sample Size		Test result	*p-value*[1]	Effect size[2]	Fig.
	HC	RRMS				HC	RRMS				
**Th17 CD4$^+$ T cells**	33	30	U = 479,5	0,835	0,029	33	28	U = 445,5	0,816	0,030	e1D
**Th22 CD4$^+$ T cells**	33	30	U = 475,5	0,792	0,040	33	28	U = 454	0,911	0,015	e1E
**ThG CD4$^+$ T cells**	33	30	U = 474,5	0,782	0,035	33	28	U = 401,000	0,384	0,113	e1F
**CCR4$^+$ Memory CD8$^+$ T cells**	33	30	U = 479,500	0,835	0,027	33	28	U = 364	0,159	0,182	e2A
**CCR6$^+$ Memory CD8$^+$ T cells**	33	30	U = 460,000	0,635	0,061	33	28	U = 321	0,041	0,261	
**CCR10$^+$ Memory CD8$^+$ T cells**	33	30	U = 426,000	0,347	0,120	33	28	U = 410	0,459	0,015	
**CxCR3$^+$ Memory CD8$^+$ T cells**	33	30	U = 398,500	0,187	0,167	33	28	U = 454	0,914	0,096	
**CCR4$^+$ CM CD8$^+$ T cells**	33	30	U = 404,500	0,216	0,157	33	28	U = 372,500	0,198	0,165	e2B
**CCR6$^+$ CM CD8$^+$ T cells**	33	30	t_{61} = 0,937	0,352	0,237	33	28	U = 394,0	0,329	0,124	
**CCR10$^+$ CM CD8$^+$ T cells**	33	30	U = 442,000	0,473	0,092	33	28	U = 401,5	0,386	0,022	
**CxCR3$^+$ CM CD8$^+$ T cells**	33	30	t_{61} = 1,248	0,217	0,315	33	28	U = 450	0,869	0,111	
**CCR4$^+$ EM CD8$^+$ T cells**	33	30	U = 460,000	0,635	0,061	33	28	U = 377	0,223	0,098	e2C
**CCR6$^+$ EM CD8$^+$ T cells**	33	30	U = 337,000	0,030	0,274	33	28	U = 409	0,450	0,157	
**CCR10$^+$ EM CD8$^+$ T cells**	33	30	U = 447,500	0,518	0,082	33	28	U = 425	0,600	0,024	
**CxCR3$^+$ EM CD8$^+$ T cells**	33	30	U = 444,500	0,492	0,087	33	28	U = 449	0,855	0,069	
**CCR4$^+$ TemRA CD8$^+$ T cells**	33	30	U = 489,000	0,937	0,010	33	28	U = 337,5	0,072	0,230	e2D
**CCR6$^+$ TemRA CD8$^+$ T cells**	33	30	t_{61} = 2,748	0,008	0,693	33	28	U = 199	0,000	0,487	
**CCR10$^+$ TemRA CD8$^+$ T cells**	33	30	U = 384,000	0,128	0,193	33	28	U = 352,5	0,114	0,084	
**CxCR3$^+$ TemRA CD8$^+$ T cells**	33	30	U = 374,500	0,098	0,209	33	28	U = 418	0,529	0,202	
**NKT cells**	22	27	U = 213,5	0,095	0,240	22	22	U = 165,0	0,072	0,272	e3A
**KLRG1$^+$ NKT cells**	21	26	U = 270,0	0,845	0,079						e3B
**NKG2A$^+$ NKT cells**	21	26	U = 257,0	0,353	0,117						e3C
**KIR2DL2/3$^+$ NKT cells**	21	26	U = 218,5	0,071	0,230						e3D
**KIR3DL1$^+$ NKT cells**	21	26	U = 195,0	0,015	0,299						e3E
**NKp30$^+$ NKT cells**	21	26	U = 263,0	0,344	0,100						e3F
**NKp44$^+$ NKT cells**	21	26	U = 263,0	0,320	0,100						e3G
**NKp46$^+$ NKT cells**	21	26	U = 223,5	0,128	0,215						e3H

[1] Red cells refere to significant differences (p-value <0.050) and grey cells to tendencies (0.050< p-value <0.010)

[2] Cohen's d was calculated for t-tests, and r for Mann-Witney U-tests. Green cells refere to large effect sizes (d>0.800; r>0.500) and yellow cells to medium effect sizes (0.300≤d≤0.800; 0.300≤r≤0.500)

CM, Central Memory; **EM**, Effector Memory; **NK**, Natural Killer; **NKT**, Natural Killer T cells; **TemRA**, Terminally differentiated CD45RA-expressing memory cells; **Th**, T helper cells; **TREC**, T cell receptor excision circles; **Treg**, Regulatory T cells.

eTable 2. Multiple linear regression models to evaluate the impact of sex (female *vs.* male), age, HCMV IgG seroprevalence and disease status (healthy *vs.* RRMS) on thymic function surrogates.

Dependent Variable	Independent Variable	Percentage of cells							Absolute number of cells							Fig
		Model	*p-value*	B	SE	*β*	*t*	p-value	Model	*p-value*	B	SE	*β*	*t*	p-value	
**CD31[+] Naive CD4[+] T cells**		F(4,34)= 12,481	**0,000**						F(4,34)= 2,774	**0,043**						1A
	Sex[1]			**-30,614**	**7,245**	**-0,580**	**-4,225**	**0,000**			-165,929	143,998	-0,219	-1,152	0,257	
	Age			-0,357	0,284	-0,177	-1,257	0,217			-9,738	5,762	-0,327	-1,690	0,100	
	HCMV IgG[2]			**9,278**	**4,262**	**0,253**	**2,177**	**0,037**			84,814	86,062	0,155	0,986	0,332	
	RRMS[3]			-5,485	3,670	-0,171	-1,495	0,144			5,567	73,426	0,012	0,076	0,940	
**sj/DJβTREC**		F(4,28)= 0,926	0,463													1B
	Sex[1]			-6,978	9,022	-0,176	-0,773	0,446								
	Age			-0,265	0,370	-0,168	-0,716	0,480								
	HCMV IgG[2]			-3,735	5,825	-0,123	-0,641	0,527								
	RRMS[3]			-1,109	5,024	-0,043	-0,221	0,827								
**sjTREC[4]**		F(4,34)= 7,866	**0,000**													1C
	Sex[1]			-141,369	139,469	-0,126	-1,014	0,316								
	Age			**-30,510**	**6,124**	**-0,603**	**-4,982**	**0,000**								
	HCMV IgG[2]			-40,483	127,629	-0,039	-0,317	0,753								
	RRMS[3]			-69,328	114,685	-0,076	-0,605	0,549								
**DJβTREC[4]**		F(4,28)= 2,627	0,056													1D
	Sex[1]			6,157	22,742	0,056	0,271	0,789								
	Age			**-2,189**	**0,932**	**-0,501**	**-2,349**	**0,026**								
	HCMV IgG[2]			-4,600	14,682	-0,055	-0,313	0,756								
	RRMS[3]			-10,597	12,663	-0,147	-0,837	0,410								

[1] Reference is "Female"; [2] Reference is "IgG negative"; [3] Reference is "Healthy"; [4] Evaluated only when at leats 10 copies were detected

HCMV, Human Cytomegalovirus; **TREC**, T cell receptor excision circles.

eTable 3. Multiple linear regression models to evaluate the impact of sex (female *vs.* male), age, HCMV IgG seroprevalence and disease status (healthy *vs.* RRMS) on $CD4^+$ T cell subsets.

Dependent Variable	Independent Variable	Percentage of cells: Model	*p-value*	B	SE	*β*	*t*	p-value	Absolute number of cells: Model	*p-value*	B	SE	*β*	*t*	p-value	Fig
**$CD4^+$ T cells**		F(4,58)= 2,804	**0,034**						F(4,56)= 1,799	0,142						2A
	Sex[1]			**-5,963**	**2,378**	**-0,313**	**-2,507**	**0,015**			**-230,602**	**105,586**	**-0,285**	**-2,184**	**0,033**	
	Age			0,262	0,135	0,247	1,937	0,058			6,088	5,930	0,136	1,027	0,309	
	HCMV IgG[2]			-1,624	2,673	-0,077	-0,607	0,546			24,684	120,488	0,027	0,205	0,838	
	RRMS[3]			3,619	2,279	0,197	1,588	0,118			-140,812	100,203	-0,181	-1,405	0,165	
**Naive/memory $CD4^+$ T cells**		F(4,58)= 3,603	**0,011**													2C
	Sex[1]			-0,269	0,279	-0,118	-0,962	0,340								
	Age			**-0,045**	**0,016**	**-0,356**	**-2,854**	**0,006**								
	HCMV IgG[2]			-0,338	0,314	-0,134	-1,076	0,286								
	RRMS[3]			-0,005	0,268	-0,002	-0,019	0,985								
**Naive $CD4^+$ T cells**		F(4,58)= 6,099	**0,000**						F(4,56)= 3,280	**0,017**						2D
	Sex[1]			**-7,157**	**3,474**	**-0,236**	**-2,060**	**0,044**			**-229,267**	**84,188**	**-0,340**	**-2,723**	**0,009**	
	Age			**-0,696**	**0,198**	**-0,412**	**-3,520**	**0,001**			-6,628	4,729	-0,178	-1,402	0,167	
	HCMV IgG[2]			-0,751	3,905	-0,022	-0,192	0,848			35,183	96,070	0,046	0,366	0,716	
	RRMS[3]			3,461	3,329	0,118	1,040	0,303			-60,447	79,896	-0,093	-0,757	0,452	
**Memory $CD4^+$ T cells**		F(4,58)= 6,099	**0,000**						F(4,56)= 4,201	**0,005**						2E
	Sex[1]			**7,157**	**3,474**	**0,236**	**2,060**	**0,044**			-1,335	60,931	-0,003	-0,022	0,983	
	Age			**0,696**	**0,198**	**0,412**	**3,520**	**0,001**			**12,716**	**3,422**	**0,460**	**3,716**	**0,000**	
	HCMV IgG[2]			0,751	3,905	0,022	0,192	0,848			-10,499	69,530	-0,018	-0,151	0,881	
	RRMS[3]			-3,461	3,329	-0,118	-1,040	0,303			-80,365	57,824	-0,167	-1,390	0,170	
**CM $CD4^+$ T cells**		F(4,58)= 1,994	0,107						F(4,56)= 1,230	0,309						2F
	Sex[1]			2,270	1,898	0,153	1,196	0,236			-26,189	39,685	-0,088	-0,660	0,512	
	Age			**0,220**	**0,108**	**0,266**	**2,033**	**0,047**			**4,776**	**2,229**	**0,290**	**2,143**	**0,036**	
	HCMV IgG[2]			-1,729	2,133	-0,105	-0,811	0,421			-42,222	45,286	-0,124	-0,932	0,355	
	RRMS[3]			0,670	1,818	0,047	0,369	0,714			-4,755	37,661	-0,017	-0,126	0,900	
**EM $CD4^+$ T cells**		F(4,58)= 6,463	**0,000**						F(4,56)= 6,160	**0,000**						2G
	Sex[1]			4,352	2,190	0,226	1,987	0,052			23,745	32,038	0,086	0,741	0,462	
	Age			**0,429**	**0,125**	**0,400**	**3,447**	**0,001**			**6,956**	**1,800**	**0,454**	**3,866**	**0,000**	
	HCMV IgG[2]			0,750	2,461	0,035	0,305	0,762			10,761	36,560	0,034	0,294	0,770	
	RRMS[3]			-3,712	2,098	-0,200	-1,769	0,082			**-68,938**	**30,405**	**-0,258**	**-2,267**	**0,027**	
**TemRA $CD4^+$ T cells**		F(4,58)= 3,775	**0,009**						F(4,56)= 4,234	**0,005**						2H
	Sex[1]			0,520	0,580	0,109	0,896	0,374			0,879	7,441	0,014	0,118	0,906	
	Age			0,047	0,033	0,178	1,437	0,156			**0,989**	**0,418**	**0,292**	**2,366**	**0,021**	
	HCMV IgG[2]			**1,723**	**0,652**	**0,327**	**2,643**	**0,011**			**20,875**	**8,491**	**0,299**	**2,459**	**0,017**	
	RRMS[3]			-0,430	0,556	-0,094	-0,774	0,442			-6,844	7,061	-0,116	-0,969	0,337	

[1] Reference is "Female"; [2] Reference is "IgG negative"; [3] Reference is "Healthy"

HCMV, Human Cytomegalovirus; **CM**, Central Memory; **EM**, Efector Memory; **TemRA**, Terminally differentiated CD45RA-expressing memory cells;

eTable 4. Multiple linear regression models to evaluate the impact of sex (female *vs.* male), age, HCMV IgG seroprevalence and disease status (healthy *vs.* RRMS) on T helper (Th) subsets.

Dependent Variable	Independent Variable	Percentage of cells							Absolute number of cells							Fig
		Model	*p-value*	B	SE	*β*	*t*	p-value	Model	*p-value*	B	SE	*β*	*t*	p-value	
**Th1**		F(4,58)= 0,900	0,470						F(4,56)= 1,455	0,228						e1A
	Sex[1]			-1,518	0,874	-0,230	-1,738	0,088			**-26,097**	**12,216**	**-0,282**	**-2,136**	**0,037**	
	Age			0,049	0,050	0,134	0,989	0,327			0,981	0,686	0,192	1,429	0,159	
	HCMV IgG[2]			0,295	0,982	0,040	0,301	0,765			3,020	13,940	0,029	0,217	0,829	
	RRMS[3]			0,042	0,837	0,007	0,051	0,960			-5,716	11,593	-0,064	-0,493	0,624	
**Th2**		F(4,58)= 6,790	**0,000**						F(4,56)= 4,028	**0,006**						e1B
	Sex[1]			**3,269**	**0,944**	**0,390**	**3,463**	**0,001**			18,106	14,084	0,157	1,286	0,204	
	Age			**0,142**	**0,054**	**0,303**	**2,637**	**0,011**			**2,387**	**0,791**	**0,375**	**3,018**	**0,004**	
	HCMV IgG[2]			-0,866	1,061	-0,093	-0,816	0,418			-12,734	16,071	-0,097	-0,792	0,431	
	RRMS[3]			-1,147	0,904	-0,142	-1,268	0,210			-22,374	13,366	-0,202	-1,674	0,100	
**Th9**		F(4,58)= 0,902	0,469						F(4,56)= 2,041	0,101						e1C
	Sex[1]			-0,836	0,507	-0,218	-1,648	0,105			**-16,815**	**6,796**	**-0,321**	**-2,474**	**0,016**	
	Age			0,038	0,029	0,179	1,323	0,191			0,723	0,382	0,249	1,893	0,064	
	HCMV IgG[2]			-0,265	0,570	-0,063	-0,465	0,643			-5,880	7,755	-0,098	-0,758	0,452	
	RRMS[3]			0,039	0,486	0,011	0,080	0,937			-4,544	6,449	-0,090	-0,705	0,484	
**Th17**		F(4,58)= 0,757	0,557						F(4,56)= 1,904	0,122						e1D
	Sex[1]			-0,376	0,278	-0,180	-1,353	0,181			**-7,753**	**3,583**	**-0,282**	**-2,164**	**0,035**	
	Age			0,013	0,016	0,114	0,841	0,404			0,255	0,201	0,168	1,267	0,210	
	HCMV IgG[2]			-0,358	0,312	-0,155	-1,145	0,257			-6,853	4,089	-0,218	-1,676	0,099	
	RRMS[3]			-0,049	0,266	-0,025	-0,186	0,853			-3,012	3,401	-0,114	-0,886	0,380	
**Th22**		F(4,58)= 1,789	0,143						F(4,56)= 2,292	0,071						e1E
	Sex[1]			**-0,509**	**0,214**	**-0,307**	**-2,381**	**0,021**			**-6,852**	**2,669**	**-0,330**	**-2,568**	**0,013**	
	Age			0,002	0,012	0,020	0,155	0,877			0,044	0,150	0,038	0,294	0,770	
	HCMV IgG[2]			-0,266	0,240	-0,145	-1,106	0,273			-4,173	3,046	-0,176	-1,370	0,176	
	RRMS[3]			-0,110	0,205	-0,069	-0,538	0,593			-2,675	2,533	-0,134	-1,056	0,295	
**ThG**		F(4,58)= 1,437	0,233						F(4,56)= 2,808	**0,034**						e1F
	Sex[1]			-0,552	0,677	-0,106	-0,816	0,418			-20,405	10,293	-0,251	-1,983	0,052	
	Age			0,064	0,038	0,221	1,659	0,103			**1,221**	**0,578**	**0,272**	**2,113**	**0,039**	
	HCMV IgG[2]			-1,448	0,760	-0,252	-1,904	0,062			**-26,612**	**11,745**	**-0,287**	**-2,266**	**0,027**	
	RRMS[3]			-0,684	0,648	-0,137	-1,055	0,296			-14,462	9,768	-0,185	-1,481	0,144	

[1] Reference is "Female"; [2] Reference is "IgG negative"; [3] Reference is "Healthy"

HCMV, Human Cytomegalovirus; **Th**, T helper cells;

eTable 5. Multiple linear regression models to evaluate the impact of sex (female *vs.* male), age, HCMV IgG seroprevalence and disease status (healthy *vs.* RRMS) on CD8+ T cell subsets.

Dependent Variable	Independent Variable	Percentage of cells: Model	*p-value*	B	SE	β	t	p-value	Absolute number of cells: Model	*p-value*	B	SE	β	t	p-value	Fig.
**CD8+ T cells**		F(4,58)= 2,816	**0,033**						F(4,56)= 2,173	0,084						3A
	Sex[1]			**5,981**	**2,376**	**0,315**	**2,517**	**0,015**			81,630	92,349	0,114	0,884	0,381	
	Age			-0,263	0,135	-0,248	-1,944	0,057			-6,106	5,187	-0,154	-1,177	0,244	
	HCMV IgG[2]			1,647	2,670	0,078	0,617	0,540			55,773	105,382	0,068	0,529	0,599	
	RRMS[3]			-3,600	2,276	-0,196	-1,581	0,119			**-211,645**	**87,641**	**-0,307**	**-2,415**	**0,019**	
**Naive/memory CD8+ T cells**		F(4,58)= 3,221	**0,019**													3C
	Sex[1]			-0,113	0,160	-0,087	-0,708	0,482								
	Age			**-0,021**	**0,009**	**-0,289**	**-2,293**	**0,025**								
	HCMV IgG[2]			-0,065	0,180	-0,046	-0,363	0,718								
	RRMS[3]			**0,317**	**0,154**	**0,254**	**2,066**	**0,043**								
**Naive CD8+ T cells**		F(4,58)= 4,059	**0,006**						F(4,56)= 1,276	0,290						3D
	Sex[1]			-5,516	3,767	-0,177	-1,464	0,148			-25,259	51,203	-0,066	-0,493	0,624	
	Age			**-0,517**	**0,214**	**-0,297**	**-2,411**	**0,019**			-5,379	2,876	-0,253	-1,870	0,067	
	HCMV IgG[2]			-0,225	4,233	-0,007	-0,053	0,958			35,440	58,429	0,081	0,607	0,547	
	RRMS[3]			**7,959**	**3,609**	**0,265**	**2,205**	**0,031**			-23,734	48,592	-0,064	-0,488	0,627	
**Memory CD8+ T cells**		F(4,58)= 4,059	**0,006**						F(4,56)= 3,539	**0,012**						3E
	Sex[1]			5,517	3,767	0,177	1,465	0,148			106,900	62,318	0,213	1,715	0,092	
	Age			**0,517**	**0,214**	**0,297**	**2,411**	**0,019**			-0,727	3,500	-0,026	-0,208	0,836	
	HCMV IgG[2]			0,226	4,233	0,007	0,053	0,958			20,336	71,113	0,035	0,286	0,776	
	RRMS[3]			**-7,960**	**3,609**	**-0,265**	**-2,206**	**0,031**			**-187,917**	**59,141**	**-0,388**	**-3,177**	**0,002**	
**CM CD8+ T cells**		F(4,58)= 2,661	**0,042**						F(4,56)= 1,133	0,351						3F
	Sex[1]			0,190	0,903	0,026	0,211	0,834			7,490	9,225	0,108	0,812	0,420	
	Age			**0,143**	**0,051**	**0,357**	**2,788**	**0,007**			0,705	0,518	0,185	1,360	0,179	
	HCMV IgG[2]			-1,530	1,015	-0,192	-1,508	0,137			-0,259	10,527	-0,003	-0,025	0,980	
	RRMS[3]			0,486	0,865	0,070	0,562	0,576			-9,376	8,755	-0,141	-1,071	0,289	
**EM CD8+ T cells**		F(4,58)= 1,809	0,139						F(4,56)= 2,555	**0,049**						3G
	Sex[1]			6,045	3,164	0,246	1,911	0,061			**93,825**	**41,703**	**0,287**	**2,250**	**0,028**	
	Age			0,161	0,180	0,117	0,892	0,376			-1,781	2,342	-0,099	-0,760	0,450	
	HCMV IgG[2]			-4,314	3,556	-0,159	-1,213	0,230			-21,887	47,589	-0,059	-0,460	0,647	
	RRMS[3]			-2,470	3,031	-0,104	-0,815	0,418			**-86,825**	**39,577**	**-0,276**	**-2,194**	**0,032**	
**TemRA CD8+ T cells**		F(4,58)= 4,449	**0,003**						F(4,56)= 2,627	**0,044**						3H
	Sex[1]			-0,716	2,554	-0,033	-0,280	0,780			5,595	35,129	0,020	0,159	0,874	
	Age			0,212	0,145	0,178	1,460	0,150			0,344	1,973	0,023	0,174	0,862	
	HCMV IgG[2]			**6,072**	**2,870**	**0,257**	**2,116**	**0,039**			42,496	40,086	0,135	1,060	0,294	
	RRMS[3]			**-5,980**	**2,447**	**-0,290**	**-2,444**	**0,018**			**-91,717**	**33,338**	**-0,345**	**-2,751**	**0,008**	

[1] Reference is "Female"; [2] Reference is "IgG negative"; [3] Reference is "Healthy"

HCMV, Human Cytomegalovirus; **CM**, Central Memory; **EM**, Effector memory; **TemRA**, Terminally differentiated CD45RA-expressing memory cells;

eTable 6. Multiple linear regression models to evaluate the impact of sex (female vs. male), age, HCMV IgG seroprevalence and disease status (healthy vs. RRMS) on chemokines receptors expressing CD8+ T cell subsets.

Dependent Variable	Independent Variable	Percentage of cells							Absolute number of cells							Fig
		Model	*p-value*	B	SE	β	*t*	p-value	Model	*p-value*	B	SE	β	*t*	p-value	
**CCR4+ Memory CD8+ T cells**		F(4,58)= 2,281	0,071						F(4,56)= 3,445	**0,014**						e2A
	Sex[1]			0,307	1,516	0,026	0,203	0,840			9,077	7,095	0,159	1,279	0,206	
	Age			**0,229**	**0,086**	**0,344**	**2,656**	**0,010**			**1,126**	**0,398**	**0,357**	**2,825**	**0,007**	
	HCMV IgG[2]			0,312	1,704	0,024	0,183	0,855			2,927	8,096	0,045	0,362	0,719	
	RRMS[3]			1,118	1,452	0,097	0,770	0,445			-4,544	6,733	-0,083	-0,675	0,503	
**CCR6+ Memory CD8+ T cells**		F(4,58)= 4,323	**0,004**						F(4,56)= 2,462	0,056						
	Sex[1]			**-6,404**	**2,064**	**-0,372**	**-3,103**	**0,003**			**-26,702**	**12,986**	**-0,263**	**-2,056**	**0,044**	
	Age			0,148	0,117	0,154	1,262	0,212			1,022	0,729	0,182	1,401	0,167	
	HCMV IgG[2]			**-6,556**	**2,319**	**-0,344**	**-2,827**	**0,006**			-24,516	14,818	-0,212	-1,654	0,104	
	RRMS[3]			0,324	1,977	0,020	0,164	0,870			**-24,982**	**12,323**	**-0,256**	**-2,027**	**0,047**	
**CCR10+ Memory CD8+ T cells**		F(4,58)= 4,690	**0,002**						F(4,56)= 3,582	**0,011**						
	Sex[1]			-0,592	0,555	-0,127	-1,067	0,290			-0,364	3,023	-0,015	-0,121	0,904	
	Age			**0,132**	**0,032**	**0,508**	**4,192**	**0,000**			**0,615**	**0,170**	**0,456**	**3,623**	**0,001**	
	HCMV IgG[2]			-0,679	0,623	-0,131	-1,089	0,281			-1,015	3,450	-0,036	-0,294	0,770	
	RRMS[3]			0,406	0,531	0,090	0,765	0,447			-1,893	2,869	-0,081	-0,660	0,512	
**CxCR3+ Memory CD8+ T cells**		F(4,58)= 1,548	0,200						F(4,56)= 0,924	0,456						
	Sex[1]			**-2,505**	**1,198**	**-0,271**	**-2,092**	**0,041**			-8,692	5,296	-0,221	-1,641	0,106	
	Age			-0,007	0,068	-0,014	-0,109	0,913			0,267	0,297	0,122	0,896	0,374	
	HCMV IgG[2]			-1,186	1,346	-0,116	-0,882	0,382			-2,130	6,043	-0,047	-0,352	0,726	
	RRMS[3]			0,539	1,147	0,061	0,470	0,640			-4,772	5,026	-0,126	-0,949	0,346	
**CCR4+ CM CD8+ T cells**		F(4,58)= 2,356	0,064						F(4,56)= 2,060	0,098						e2B
	Sex[1]			2,700	2,843	0,120	0,950	0,346			3,735	3,462	0,140	1,079	0,285	
	Age			**0,371**	**0,162**	**0,296**	**2,292**	**0,026**			**0,400**	**0,194**	**0,271**	**2,055**	**0,045**	
	HCMV IgG[2]			1,586	3,195	0,064	0,496	0,621			2,630	3,950	0,086	0,666	0,508	
	RRMS[3]			-1,534	2,723	-0,071	-0,563	0,575			0,047	3,285	0,002	0,014	0,989	
**CCR6+ CM CD8+ T cells**		F(4,58)= 5,716	**0,001**						F(4,56)= 3,878	**0,008**						
	Sex[1]			**-2,515**	**1,056**	**-0,275**	**-2,381**	**0,021**			**-1,998**	**0,810**	**-0,303**	**-2,467**	**0,017**	
	Age			-0,074	0,060	-0,145	-1,226	0,225			0,017	0,045	0,046	0,366	0,716	
	HCMV IgG[2]			**-3,783**	**1,187**	**-0,374**	**-3,186**	**0,002**			**-2,515**	**0,924**	**-0,334**	**-2,721**	**0,009**	
	RRMS[3]			0,243	1,012	0,028	0,240	0,811			-1,510	0,769	-0,238	-1,965	0,054	
**CCR10+ CM CD8+ T cells**		F(4,58)= 3,178	**0,020**						F(4,56)= 2,242	0,076						
	Sex[1]			-1,512	1,050	-0,178	-1,440	0,155			-0,462	1,107	-0,054	-0,417	0,678	
	Age			**0,210**	**0,060**	**0,443**	**3,508**	**0,001**			**0,183**	**0,062**	**0,385**	**2,941**	**0,005**	
	HCMV IgG[2]			-0,744	1,180	-0,079	-0,630	0,531			-0,178	1,263	-0,018	-0,141	0,888	
	RRMS[3]			-0,521	1,006	-0,064	-0,518	0,607			-0,121	1,050	-0,015	-0,115	0,909	
**CxCR3+ CM CD8+ T cells**		F(4,58)= 1,235	0,306						F(4,56)= 0,718	0,583						
	Sex[1]			-6,481	3,817	-0,223	-1,698	0,095			-4,536	3,414	-0,180	-1,329	0,189	
	Age			0,016	0,217	0,010	0,072	0,943			0,256	0,192	0,183	1,333	0,188	
	HCMV IgG[2]			-2,531	4,290	-0,079	-0,590	0,557			-1,075	3,895	-0,037	-0,276	0,784	
	RRMS[3]			3,904	3,657	0,139	1,068	0,290			-1,246	3,240	-0,051	-0,385	0,702	
**CCR4+ EM CD8+ T cells**		F(4,58)= 5,047	**0,001**						F(4,56)= 4,927	**0,002**						e2C
	Sex[1]			0,020	1,285	0,002	0,015	0,988			4,751	3,554	0,160	1,337	0,187	
	Age			**0,303**	**0,073**	**0,497**	**4,143**	**0,000**			**0,700**	**0,200**	**0,425**	**3,507**	**0,001**	
	HCMV IgG[2]			0,522	1,444	0,043	0,361	0,719			-0,384	4,056	-0,011	-0,095	0,925	
	RRMS[3]			0,052	1,231	0,005	0,042	0,967			-4,322	3,373	-0,151	-1,281	0,205	
**CCR6+ EM CD8+ T cells**		F(4,58)= 6,089	**0,000**						F(4,56)= 3,400	**0,015**						
	Sex[1]			**-8,823**	**2,810**	**-0,360**	**-3,140**	**0,003**			**-22,271**	**9,472**	**-0,293**	**-2,351**	**0,022**	
	Age			0,176	0,160	0,129	1,100	0,276			0,604	0,532	0,144	1,135	0,261	
	HCMV IgG[2]			**-10,056**	**3,158**	**-0,371**	**-3,184**	**0,002**			**-30,853**	**10,808**	**-0,355**	**-2,855**	**0,006**	
	RRMS[3]			3,989	2,692	0,169	1,482	0,144			0,667	8,989	0,009	0,074	0,941	

[1] Reference is "Female"; [2] Reference is "IgG negative"; [3] Reference is "Healthy"

HCMV, Human Cytomegalovirus; **CM**, Central Memory; **EM**, Efector Memory; **TemRA**, Terminally differentiated CD45RA-expressing memory cells;

eTable 6 (cont.)

Dependent Variable	Independent Variable	Percentage of cells: Model	*p-value*	B	SE	*β*	*t*	p-value	Absolute number of cells: Model	*p-value*	B	SE	*β*	*t*	p-value	Fig
**CCR10⁺ EM CD8⁺ T cells**		F(4,58)= 7,920	**0,000**						F(4,56)= 4,375	**0,004**						e2C
	Sex[1]			-0,740	0,550	-0,148	-1,346	0,184			-0,614	1,841	-0,040	-0,333	0,740	
	Age			**0,174**	**0,031**	**0,623**	**5,555**	**0,000**			**0,423**	**0,103**	**0,504**	**4,094**	**0,000**	
	HCMV IgG[2]			-0,402	0,618	-0,073	-0,651	0,517			-1,268	2,101	-0,073	-0,603	0,549	
	RRMS[3]			0,273	0,527	0,057	0,518	0,606			-0,866	1,747	-0,059	-0,495	0,622	
**CxCR3⁺ EM CD8⁺ T cells**		F(4,58)= 0,801	0,530						F(4,56)= 0,823	0,516						
	Sex[1]			-1,401	0,845	-0,220	-1,658	0,103			-2,390	2,026	-0,159	-1,180	0,243	
	Age			0,046	0,048	0,130	0,957	0,343			0,134	0,114	0,162	1,181	0,243	
	HCMV IgG[2]			0,128	0,950	0,018	0,135	0,893			-0,208	2,312	-0,012	-0,090	0,928	
	RRMS[3]			-0,142	0,810	-0,023	-0,175	0,862			-2,067	1,923	-0,143	-1,075	0,287	
**CCR4⁺ TemRA CD8⁺ T cells**		F(4,58)= 0,377	0,824						F(4,56)= 1,042	0,394						e2D
	Sex[1]			-0,144	0,532	-0,037	-0,271	0,787			0,294	0,491	0,080	0,599	0,552	
	Age			0,006	0,030	0,028	0,206	0,837			0,036	0,028	0,176	1,291	0,202	
	HCMV IgG[2]			0,297	0,597	0,068	0,497	0,621			0,244	0,560	0,058	0,436	0,665	
	RRMS[3]			0,582	0,509	0,153	1,144	0,257			-0,442	0,466	-0,125	-0,949	0,347	
**CCR6⁺ TemRA CD8⁺ T cells**		F(4,58)= 3,949	**0,007**						F(4,56)= 3,788	**0,009**						
	Sex[1]			-1,563	1,877	-0,101	-0,832	0,409			-2,595	7,543	-0,042	-0,344	0,732	
	Age			**0,294**	**0,107**	**0,340**	**2,749**	**0,008**			0,385	0,424	0,114	0,908	0,368	
	HCMV IgG[2]			-0,595	2,110	-0,035	-0,282	0,779			8,152	8,608	0,117	0,947	0,348	
	RRMS[3]			**-5,086**	**1,799**	**-0,340**	**-2,828**	**0,006**			**-24,067**	**7,159**	**-0,408**	**-3,362**	**0,001**	
**CCR10⁺ TemRA CD8⁺ T cells**		F(4,58)= 0,410	0,801						F(4,56)= 0,673	0,613						
	Sex[1]			0,298	0,367	0,109	0,812	0,420			0,762	0,848	0,122	0,899	0,372	
	Age			-0,016	0,021	-0,108	-0,785	0,436			0,010	0,048	0,030	0,217	0,829	
	HCMV IgG[2]			-0,234	0,413	-0,077	-0,566	0,573			0,417	0,967	0,058	0,431	0,668	
	RRMS[3]			0,034	0,352	0,013	0,096	0,924			-0,867	0,804	-0,144	-1,078	0,286	
**CxCR3⁺ TemRA CD8⁺ T cells**		F(4,58)= 4,290	**0,004**						F(4,56)= 1,858	0,131						
	Sex[1]			-0,931	0,471	-0,237	-1,977	0,053			**-0,949**	**0,408**	**-0,303**	**-2,327**	**0,024**	
	Age			-0,041	0,027	-0,187	-1,530	0,131			-0,006	0,023	-0,034	-0,255	0,800	
	HCMV IgG[2]			**-1,154**	**0,529**	**-0,266**	**-2,180**	**0,033**			-0,359	0,465	-0,101	-0,771	0,444	
	RRMS[3]			0,398	0,451	0,105	0,883	0,381			-0,325	0,387	-0,108	-0,839	0,405	

[1] Reference is "Female"; [2] Reference is "IgG negative"; [3] Reference is "Healthy"

HCMV, Human Cytomegalovirus; **CM**, Central Memory; **EM**, Efector Memory; **TemRA**, Terminally differentiated CD45RA-expressing memory cells;

eTable 7. Multiple linear regression models to evaluate the impact of sex (female *vs.* male), age, HCMV IgG seroprevalence and disease status (healthy *vs.* RRMS) on regulatory T cells (Treg) subsets.

Dependent Variable	Independent Variable	Percentage of cells: Model	*p-value*	B	SE	β	*t*	p-value	Absolute number of cells: Model	*p-value*	B	SE	β	*t*	p-value	Fig.
**Treg**		F(4,58)= 1,791	0,143						F(4,56)= 0,356	0,839						4A
	Sex[1]			0,898	0,471	0,245	1,906	0,062			-1,526	8,599	-0,024	-0,178	0,860	
	Age			-0,017	0,027	-0,086	-0,652	0,517			0,351	0,483	0,101	0,727	0,470	
	HCMV IgG[2]			-0,598	0,530	-0,148	-1,129	0,264			-8,837	9,812	-0,123	-0,901	0,372	
	RRMS[3]			0,451	0,452	0,128	0,999	0,322			2,597	8,160	0,043	0,318	0,751	
**Naive/Activated Tregs[4]**		F(4,58)= 4,638	**0,003**													4C
	Sex[1]			0,116	0,090	0,154	1,295	0,200								
	Age			**-0,019**	**0,005**	**-0,445**	**-3,663**	**0,001**								
	HCMV IgG[2]			-0,015	0,101	-0,018	-0,150	0,881								
	RRMS[3]			0,166	0,086	0,227	1,923	0,059								
**CD45RA$^+$HLA-DR$^-$ Tregs[4]**		F(4,58)= 5,688	**0,001**						F(4,56)= 1,950	0,115						4D
	Sex[1]			4,905	2,938	0,193	1,670	0,100			3,945	3,407	0,151	1,158	0,252	
	Age			**-0,666**	**0,167**	**-0,471**	**-3,986**	**0,000**			**-0,432**	**0,191**	**-0,299**	**-2,260**	**0,028**	
	HCMV IgG[2]			3,348	3,301	0,119	1,014	0,315			0,034	3,888	0,001	0,009	0,993	
	RRMS[3]			**7,635**	**2,814**	**0,312**	**2,713**	**0,009**			4,917	3,234	0,195	1,520	0,134	
**CD45RA$^-$HLA-DR$^-$ Tregs[4]**		F(4,58)= 4,055	**0,006**						F(4,56)= 1,107	0,362						4E
	Sex[1]			**-6,167**	**2,083**	**-0,357**	**-2,961**	**0,004**			-5,240	4,282	-0,164	-1,224	0,226	
	Age			**0,291**	**0,118**	**0,302**	**2,452**	**0,017**			0,440	0,240	0,249	1,830	0,073	
	HCMV IgG[2]			0,237	2,341	0,012	0,101	0,920			-5,002	4,886	-0,137	-1,024	0,310	
	RRMS[3]			-3,839	1,996	-0,231	-1,924	0,059			-0,746	4,063	-0,024	-0,184	0,855	
**CD45RA$^-$HLA-DR$^+$ Tregs[4]**		F(4,58)= 3,577	**0,011**						F(4,56)= 1,969	0,112						4F
	Sex[1]			1,128	2,219	0,062	0,508	0,613			-0,349	2,448	-0,019	-0,143	0,887	
	Age			**0,383**	**0,126**	**0,379**	**3,033**	**0,004**			**0,350**	**0,137**	**0,336**	**2,542**	**0,014**	
	HCMV IgG[2]			-3,692	2,494	-0,184	-1,480	0,144			-3,934	2,793	-0,183	-1,408	0,165	
	RRMS[3]			-3,995	2,126	-0,228	-1,879	0,065			-1,749	2,323	-0,096	-0,753	0,455	
**CD39$^+$ Tregs[4]**		F(4,58)= 2,737	**0,037**						F(4,56)= 2,315	0,068						4G
	Sex[1]			-2,944	3,739	-0,099	-0,787	0,434			-3,489	3,757	-0,119	-0,929	0,357	
	Age			**0,513**	**0,213**	**0,308**	**2,412**	**0,019**			**0,524**	**0,211**	**0,324**	**2,482**	**0,016**	
	HCMV IgG[2]			-5,834	4,201	-0,177	-1,389	0,170			-7,953	4,287	-0,238	-1,855	0,069	
	RRMS[3]			**-8,448**	**3,582**	**-0,294**	**-2,359**	**0,022**			-5,064	3,566	-0,180	-1,420	0,161	
**CD73$^+$ Tregs[4]**		F(4,58)= 0,500	0,736						F(4,56)= 1,303	0,280						4H
	Sex[1]			-0,752	0,696	-0,145	-1,080	0,284			-0,928	0,537	-0,229	-1,727	0,090	
	Age			-0,015	0,040	-0,053	-0,389	0,699			0,013	0,030	0,059	0,441	0,661	
	HCMV IgG[2]			-0,373	0,782	-0,065	-0,478	0,635			-0,936	0,613	-0,203	-1,527	0,132	
	RRMS[3]			-0,147	0,666	-0,029	-0,220	0,827			-0,281	0,510	-0,072	-0,551	0,584	
**GARP$^+$ Tregs[4]**		F(4,58)= 2,285	0,071						F(4,56)= 1,560	0,198						4I
	Sex[1]			1,158	1,223	0,120	0,947	0,348			1,294	1,428	0,119	0,906	0,369	
	Age			-0,071	0,070	-0,133	-1,027	0,309			-0,051	0,080	-0,085	-0,635	0,528	
	HCMV IgG[2]			2,305	1,374	0,216	1,678	0,099			1,782	1,629	0,144	1,094	0,279	
	RRMS[3]			**3,126**	**1,171**	**0,337**	**2,669**	**0,010**			**3,066**	**1,355**	**0,294**	**2,263**	**0,028**	

[1] Reference is "Female"; [2] Reference is "IgG negative"; [3] Reference is "Healthy"; [4] Evaluated only when parent population >500 events

HCMV, Human Cytomegalovirus; **Treg**, Regulatory T cells

eTable 8. Multiple linear regression models to evaluate the impact of sex (female *vs.* male), age, HCMV IgG seroprevalence and disease status (healthy *vs.* RRMS) on natural killer (NK) cells subsets.

Dependent Variable	Independent Variable	Percentage of cells: Model	*p-value*	B	SE	*β*	*t*	p-value	Absolute number of cells: Model	*p-value*	B	SE	*β*	*t*	p-value	Fig
**NK cells**		F(4,43)= 1,502	0,219						F(4,38)= 0,795	0,536						5A
	Sex[1]			4,084	2,404	0,258	1,698	0,097			116,994	124,290	0,157	0,941	0,352	
	Age			0,116	0,106	0,163	1,096	0,279			5,612	5,290	0,171	1,061	0,296	
	HCMV IgG[2]			-1,925	2,200	-0,133	-0,875	0,387			-47,098	113,728	-0,068	-0,414	0,681	
	RRMS[3]			-0,343	1,977	-0,027	-0,173	0,863			32,989	102,687	0,054	0,321	0,750	
**CD56^{bright} NK cells**		F(4,43)= 2,806	**0,037**						F(4,38)= 1,377	0,260						5B
	Sex[1]			-1,984	1,629	-0,176	-1,218	0,230			-6,902	4,811	-0,233	-1,435	0,160	
	Age			**-0,165**	**0,072**	**-0,325**	**-2,303**	**0,026**			-0,189	0,205	-0,144	-0,922	0,362	
	HCMV IgG[2]			1,823	1,491	0,177	1,223	0,228			0,550	4,402	0,020	0,125	0,901	
	RRMS[3]			2,508	1,340	0,273	1,872	0,068			6,721	3,974	0,279	1,691	0,099	
**CD56^{dim}CD57$^-$ NK cells**		F(4,43)= 1,971	0,116						F(4,38)= 1,068	0,386						5C
	Sex[1]			-2,713	4,467	-0,091	-0,607	0,547			18,965	59,938	0,052	0,316	0,753	
	Age			0,011	0,196	0,008	0,057	0,954			2,834	2,551	0,176	1,111	0,274	
	HCMV IgG[2]			**-10,633**	**4,087**	**-0,388**	**-2,601**	**0,013**			-78,910	54,845	-0,233	-1,439	0,158	
	RRMS[3]			0,926	3,673	0,038	0,252	0,802			23,535	49,520	0,080	0,475	0,637	
**CD56^{dim}CD57$^+$ NK cells**		F(4,43)= 1,856	0,136						F(4,38)= 1,227	0,316						5D
	Sex[1]			4,802	4,970	0,145	0,966	0,339			104,821	67,323	0,255	1,557	0,128	
	Age			0,159	0,218	0,107	0,728	0,470			2,968	2,866	0,163	1,036	0,307	
	HCMV IgG[2]			8,620	4,548	0,284	1,895	0,065			30,776	61,602	0,080	0,500	0,620	
	RRMS[3]			-3,599	4,087	-0,133	-0,881	0,383			2,247	55,621	0,007	0,040	0,968	
**KLRG1$^+$ CD56^{bright} NK cells[4]**		F(4,33)= 3,949	**0,010**													5E
	Sex[1]			**8,124**	**2,935**	**0,418**	**2,768**	**0,009**								
	Age			-0,054	0,132	-0,060	-0,410	0,684								
	HCMV IgG[2]			0,137	2,707	0,008	0,051	0,960								
	RRMS[3]			**5,128**	**2,429**	**0,322**	**2,111**	**0,042**								
**KLRG1$^+$ CD56^{dim}CD57$^-$ NK cells[4]**		F(4,41)= 0,031	0,998													
	Sex[1]			-1,914	6,880	-0,046	-0,278	0,782								
	Age			0,066	0,299	0,036	0,220	0,827								
	HCMV IgG[2]			-0,319	6,390	-0,008	-0,050	0,960								
	RRMS[3]			-0,415	5,585	-0,013	-0,074	0,941								
**KLRG1$^+$ CD56^{dim}CD57$^+$ NK cells[4]**		F(4,41)= 0,678	0,611													
	Sex[1]			-3,575	8,479	-0,068	-0,422	0,675								
	Age			0,206	0,368	0,088	0,558	0,580								
	HCMV IgG[2]			7,798	7,875	0,158	0,990	0,328								
	RRMS[3]			-3,915	6,883	-0,093	-0,569	0,573								
**NKG2A$^+$ CD56^{bright} NK cells[4]**		F(4,33)= 1,086	0,380													5F
	Sex[1]			-1,066	1,279	-0,144	-0,834	0,410								
	Age			-0,037	0,058	-0,107	-0,636	0,529								
	HCMV IgG[2]			-0,956	1,180	-0,139	-0,811	0,423								
	RRMS[3]			1,439	1,059	0,237	1,359	0,183								
**NKG2A$^+$ CD56^{dim}CD57$^-$ NK cells[4]**		F(4,41)= 1,387	0,255													
	Sex[1]			-1,148	4,688	-0,038	-0,245	0,808								
	Age			-0,128	0,204	-0,095	-0,627	0,534								
	HCMV IgG[2]			-4,799	4,354	-0,171	-1,102	0,277								
	RRMS[3]			5,508	3,805	0,229	1,447	0,155								

[1] Reference is "Female"; [2] Reference is "IgG negative"; [3] Reference is "Healthy"; [4] Evaluated only when parent population >500 events

HCMV, Human Cytomegalovirus; **NK**, Natural Killer.

eTable 8 (cont.)

Dependent Variable	Independent Variable	Percentage of cells: Model	*p-value*	B	SE	*β*	*t*	p-value	Absolute number of cells: Model	*p-value*	B	SE	*β*	*t*	p-value	Fig.
**NKG2A^{+} CD56^{dim}CD57^{+} NK cells[4]**		F(4,41)= 3,827	**0,010**													5F
	Sex[1]			-10,285	6,479	-0,224	-1,587	0,120								
	Age			0,132	0,281	0,065	0,468	0,642								
	HCMV IgG[2]			**-17,651**	**6,018**	**-0,414**	**-2,933**	**0,005**								
	RRMS[3]			7,960	5,259	0,218	1,514	0,138								
**KIR2DL2/3^{+} CD56^{bright} NK cells[4]**		F(4,33)= 0,069	0,991													5G
	Sex[1]			0,183	0,526	0,064	0,347	0,730								
	Age			0,002	0,024	0,012	0,067	0,947								
	HCMV IgG[2]			0,024	0,485	0,009	0,050	0,960								
	RRMS[3]			-0,173	0,436	-0,073	-0,397	0,694								
**KIR2DL2/3^{+} CD56^{dim}CD57^{-} NK cells[4]**		F(4,41)= 0,827	0,516													
	Sex[1]			0,320	1,458	0,035	0,220	0,827								
	Age			0,089	0,063	0,219	1,405	0,167								
	HCMV IgG[2]			0,990	1,354	0,116	0,731	0,469								
	RRMS[3]			-0,248	1,183	-0,034	-0,210	0,835								
**KIR2DL2/3^{+} CD56^{dim}CD57^{+} NK cells[4]**		F(4,41)= 0,213	0,930													
	Sex[1]			-0,738	3,087	-0,039	-0,239	0,812								
	Age			0,101	0,134	0,120	0,751	0,457								
	HCMV IgG[2]			1,147	2,867	0,065	0,400	0,691								
	RRMS[3]			0,050	2,506	0,003	0,020	0,984								
**KIR3DL1^{+} CD56^{bright} NK cells[4]**		F(4,33)= 0,919	0,464													5H
	Sex[1]			1,285	1,055	0,212	1,218	0,232								
	Age			-0,034	0,048	-0,120	-0,708	0,484								
	HCMV IgG[2]			-1,063	0,973	-0,190	-1,092	0,283								
	RRMS[3]			0,114	0,874	0,023	0,130	0,897								
**KIR3DL1^{+} CD56^{dim}CD57^{-} NK cells[4]**		F(4,41)= 0,792	0,537													
	Sex[1]			6,673	4,327	0,246	1,542	0,131								
	Age			-0,081	0,188	-0,067	-0,431	0,669								
	HCMV IgG[2]			-2,795	4,019	-0,111	-0,695	0,491								
	RRMS[3]			-3,290	3,512	-0,152	-0,937	0,354								
**KIR3DL1^{+} CD56^{dim}CD57^{+} NK cells[4]**		F(4,41)= 1,582	0,197													
	Sex[1]			8,986	5,159	0,268	1,742	0,089								
	Age			-0,079	0,224	-0,053	-0,354	0,725								
	HCMV IgG[2]			-7,838	4,791	-0,252	-1,636	0,110								
	RRMS[3]			-5,655	4,187	-0,212	-1,350	0,184								
**NKp30^{+} CD56^{bright} NK cells**		F(4,27)= 1,750	0,168													5I
	Sex[1]			1,667	3,101	0,108	0,537	0,595								
	Age			0,231	0,150	0,307	1,538	0,136								
	HCMV IgG[2]			-3,214	2,463	-0,252	-1,305	0,203								
	RRMS[3]			0,964	2,267	0,080	0,425	0,674								

[1] Reference is "Female"; [2] Reference is "IgG negative"; [3] Reference is "Healthy"; [4] Evaluated only when parent population >500 events

HCMV, Human Cytomegalovirus; **NK**, Natural Killer.

eTable 8 (cont.)

Dependent Variable	Independent Variable	Percentage of cells: Model	*p-value*	B	SE	*β*	*t*	*p-value*	Absolute number of cells: Model	*p-value*	B	SE	*β*	*t*	*p-value*	Fig.
**NKp30$^+$ CD56^{dim}CD57$^-$ NK cells**[4]		F(4,43)= 1,126	0,357													5I
	Sex[1]			1,643	2,252	0,113	0,730	0,470								
	Age			0,176	0,099	0,268	1,780	0,082								
	HCMV IgG[2]			-1,096	2,061	-0,082	-0,532	0,598								
	RRMS[3]			-0,331	1,852	-0,028	-0,179	0,859								
**NKp30$^+$ CD56^{dim}CD57$^+$ NK cells**[4]		F(4,41)= 2,444	0,061													
	Sex[1]			-0,067	1,468	-0,007	-0,046	0,964								
	Age			0,034	0,063	0,079	0,545	0,589								
	HCMV IgG[2]			-1,858	1,307	-0,207	-1,421	0,163								
	RRMS[3]			**2,631**	**1,159**	**0,336**	**2,271**	**0,028**								
**NKp44$^+$ CD56^{bright} NK cells**[4]		F(4,27)= 0,726	0,582													5J
	Sex[1]			4,522	4,096	0,236	1,104	0,279								
	Age			-0,151	0,199	-0,162	-0,762	0,453								
	HCMV IgG[2]			-2,908	3,253	-0,184	-0,894	0,379								
	RRMS[3]			-0,839	2,994	-0,056	-0,280	0,782								
**NKp44$^+$ CD56^{dim}CD57$^-$ NK cells**[4]		F(4,43)= 0,638	0,638													
	Sex[1]			2,013	2,087	0,152	0,964	0,340								
	Age			-0,119	0,092	-0,199	-1,295	0,202								
	HCMV IgG[2]			-0,144	1,910	-0,012	-0,075	0,940								
	RRMS[3]			-1,331	1,717	-0,123	-0,775	0,443								
**NKp44$^+$ CD56^{dim}CD57$^+$ NK cells**[4]		F(4,41)= 0,326	0,859													
	Sex[1]			0,554	3,389	0,026	0,163	0,871								
	Age			-0,019	0,145	-0,021	-0,134	0,894								
	HCMV IgG[2]			0,093	3,019	0,005	0,031	0,975								
	RRMS[3]			-2,928	2,675	-0,177	-1,094	0,280								
**NKp46$^+$ CD56^{bright} NK cells**[4]		F(4,27)= 0,499	0,736													5K
	Sex[1]			0,693	1,214	0,124	0,571	0,573								
	Age			0,014	0,059	0,052	0,239	0,813								
	HCMV IgG[2]			-0,563	0,964	-0,122	-0,584	0,564								
	RRMS[3]			0,483	0,887	0,110	0,544	0,591								
**NKp46$^+$ CD56^{dim}CD57$^-$ NK cells**[4]		F(4,43)= 0,779	0,545													
	Sex[1]			-5,732	4,363	-0,206	-1,314	0,196								
	Age			-0,002	0,192	-0,001	-0,008	0,993								
	HCMV IgG[2]			-1,314	3,993	-0,052	-0,329	0,744								
	RRMS[3]			-2,831	3,588	-0,125	-0,789	0,434								
**NKp46$^+$ CD56^{dim}CD57$^+$ NK cells**[4]		F(4,41)= 2,030	0,108													
	Sex[1]			-4,913	5,387	-0,137	-0,912	0,367								
	Age			-0,017	0,230	-0,011	-0,073	0,942								
	HCMV IgG[2]			**-11,818**	**4,799**	**-0,365**	**-2,463**	**0,018**								
	RRMS[3]			2,339	4,253	0,083	0,550	0,585								

[1] Reference is "Female"; [2] Reference is "IgG negative"; [3] Reference is "Healthy"; [4] Evaluated only when parent population >500 events

HCMV, Human Cytomegalovirus; **NK**, Natural Killer.

eTable 9. Multiple linear regression models to evaluate the impact of sex (female *vs.* male), age, HCMV IgG seroprevalence and disease status (healthy *vs.* RRMS) on NKT cells.

Dependent Variable	Independent Variable	Percentage of cells: Model	p-value	B	SE	β	t	*p-value*	Absolute number of cells: Model	p-value	B	SE	β	t	p-value	Fig.
**NKT cells**		F(4,43)= 1,485	0,224						F(4,38)= 0,746	0,576						e3A
	Sex[1]			0,091	0,831	0,017	0,109	0,914			32,201	36,008	0,150	0,894	0,377	
	Age			-0,009	0,037	-0,036	-0,241	0,811			-0,040	1,533	-0,004	-0,026	0,979	
	HCMV IgG[2]			**1,799**	**0,761**	**0,360**	**2,364**	**0,023**			48,190	32,948	0,240	1,463	0,152	
	RRMS[3]			0,081	0,684	0,018	0,118	0,907			1,196	29,749	0,007	0,040	0,968	
**KLRG1+ NKT cells**		F(4,41)= 0,862	0,495													e3B
	Sex[1]			1,805	3,147	0,091	0,574	0,569								
	Age			-0,097	0,137	-0,111	-0,713	0,480								
	HCMV IgG[2]			-3,842	2,923	-0,209	-1,314	0,196								
	RRMS[3]			-3,543	2,554	-0,225	-1,387	0,173								
**NKG2A+ NKT cells**		F(4,41)= 4,127	**0,007**													e3C
	Sex[1]			**-10,597**	**5,153**	**-0,287**	**-2,056**	**0,046**								
	Age			0,103	0,224	0,063	0,462	0,647								
	HCMV IgG[2]			**-17,222**	**4,786**	**-0,502**	**-3,598**	**0,001**								
	RRMS[3]			-1,336	4,183	-0,045	-0,319	0,751								
**KIR2DL2/3+ NKT cells**		F(4,41)= 1,630	0,185													e3D
	Sex[1]			-1,691	3,647	-0,071	-0,464	0,645								
	Age			-0,069	0,158	-0,065	-0,434	0,666								
	HCMV IgG[2]			4,568	3,388	0,207	1,348	0,185								
	RRMS[3]			-4,190	2,961	-0,222	-1,415	0,165								
**KIR3DL1+ NKT cells**		F(4,41)= 2,539	0,054													e3E
	Sex[1]			-0,637	2,969	-0,032	-0,215	0,831								
	Age			-0,194	0,129	-0,218	-1,502	0,141								
	HCMV IgG[2]			**6,073**	**2,757**	**0,326**	**2,203**	**0,033**								
	RRMS[3]			-2,520	2,410	-0,158	-1,046	0,302								
**NKp30+ NKT cells**		F(4,41)= 2,762	**0,040**													e3F
	Sex[1]			-3,772	3,406	-0,163	-1,107	0,275								
	Age			-0,281	0,146	-0,278	-1,922	0,062								
	HCMV IgG[2]			-4,633	3,032	-0,221	-1,528	0,134								
	RRMS[3]			2,596	2,707	0,141	0,959	0,343								
**NKp44+ NKT cells**		F(4,41)= 0,300	0,876													e3G
	Sex[1]			2,760	2,877	0,157	0,959	0,343								
	Age			-0,067	0,123	-0,088	-0,547	0,587								
	HCMV IgG[2]			0,570	2,561	0,036	0,222	0,825								
	RRMS[3]			-1,292	2,287	-0,092	-0,565	0,575								
**NKp46+ NKT cells**		F(4,41)= 1,267	0,298													e3H
	Sex[1]			0,166	0,786	0,033	0,212	0,834								
	Age			-0,043	0,034	-0,196	-1,272	0,211								
	HCMV IgG[2]			0,760	0,699	0,167	1,087	0,283								
	RRMS[3]			1,071	0,624	0,268	1,716	0,094								

[1] Reference is "Female"; [2] Reference is "IgG negative"; [3] Reference is "Healthy"

eTable 10. Multiple linear regression models to evaluate the impact of time since last relapse on the several blood cell populations while controlling for Corticosteroids administration to treat the relapse. The unstandardized residuals from a preliminary multiple linear regression were calculated, where the contribution of sex, age and HCMV IgG seroprevalence on the blood populations was evaluated. The residuals were used as dependent variable to evaluate the impact of time since last relapse and corticosteroids on the blood cell populations. Only the blood populations for which the multiple linear regressions were significant ($p < 0.050$) for time from relapse are represented.

Dependent Variable	Independent Variable	Percentage of cells								Absolute number of cells							
		Model	p-value	Effect Size (R^2)	B	SE	*β*	*t*	*p-value*	Model	p-value	Effect Size (R^2)	B	SE	*β*	*t*	*p-value*
**CD4$^+$ T cells**										F(2,24)= 7,857	**0,002**	*0,396*					
	Last relapse (months)												**26,244**	**6,620**	**0,636**	**3,964**	**0,001**
	Corticosteroids[1]												86,418	145,155	0,096	0,595	0,557
**Naive CD4$^+$ T cells**										F(2,24)= 6,388	**0,006**	*0,347*					
	Last relapse (months)												**14,903**	**4,170**	**0,596**	**3,574**	**0,002**
	Corticosteroids[1]												46,422	91,422	0,085	0,508	0,616
**Memory CD4$^+$ T cells**										F(2,24)= 4,337	**0,025**	*0,265*					
	Last relapse (months)												**11,340**	**3,851**	**0,521**	**2,945**	**0,007**
	Corticosteroids[1]												39,995	84,435	0,084	0,474	0,640
**CM CD4$^+$ T cells**										F(2,24)= 5,568	**0,010**	*0,317*					
	Last relapse (months)												**9,218**	**2,887**	**0,545**	**3,192**	**0,004**
	Corticosteroids[1]												-30,623	63,309	-0,083	-0,484	0,633
**Th2**										F(2,24)= 4,298	**0,025**	*0,264*					
	Last relapse (months)												**2,203**	**0,914**	**0,427**	**2,410**	**0,024**
	Corticosteroids[1]												-25,869	20,042	-0,229	-1,291	0,209
**CM CD8$^+$ T cells**		F(2,26)= 6,771	**0,004**	0,342						F(2,24)= 5,506	**0,011**	0,315					
	Last relapse (months)				**0,109**	**0,045**	**0,386**	**2,402**	**0,024**				**1,634**	**0,496**	**0,562**	**3,291**	**0,003**
	Corticosteroids[1]				**-2,316**	**0,965**	**-0,386**	**-2,399**	**0,024**				0,758	10,886	0,012	0,070	0,945
**CCR4$^+$ Memory CD8$^+$ T cells**		F(2,24)= 4,797	**0,017**	0,270						F(2,24)= 5,544	**0,010**	0,316					
	Last relapse (months)				**0,148**	**0,056**	**0,450**	**2,652**	**0,013**				**1,019**	**0,320**	**0,544**	**3,183**	**0,004**
	Corticosteroids[1]				-1,415	1,192	-0,201	-1,187	0,246				-3,442	7,021	-0,084	-0,490	0,628
**CCR6$^+$ Memory CD8$^+$ T cells**		F(2,24)= 6,732	**0,004**	0,341													
	Last relapse (months)				**-0,407**	**0,137**	**-0,479**	**-2,975**	**0,006**								
	Corticosteroids[1]				4,908	2,920	0,271	1,681	0,105								
**CCR10$^+$ Memory CD8$^+$ T cells**		F(2,24)= 3,631	**0,041**	0,218						F(2,24)= 5,179	**0,013**	0,301					
	Last relapse (months)				**0,073**	**0,028**	**0,459**	**2,620**	**0,014**				**0,478**	**0,149**	**0,555**	**3,218**	**0,004**
	Corticosteroids[1]				-0,140	0,596	-0,041	-0,235	0,816				1,702	3,258	0,090	0,522	0,606
**CCR4$^+$ CM CD8$^+$ T cells**		F(2,24)= 6,948	**0,004**	0,348						F(2,24)= 8,335	**0,002**	0,410					
	Last relapse (months)				**0,360**	**0,107**	**0,537**	**3,354**	**0,002**				**0,614**	**0,157**	**0,622**	**3,923**	**0,001**
	Corticosteroids[1]				-2,535	2,286	-0,178	-1,109	0,278				-1,829	3,434	-0,084	-0,532	0,599
**CCR6$^+$ CM CD8$^+$ T cells**		F(2,24)= 5,098	**0,014**	0,282													
	Last relapse (months)				**-0,196**	**0,071**	**-0,461**	**-2,743**	**0,011**								
	Corticosteroids[1]				1,838	1,522	0,203	1,208	0,238								
**CCR10$^+$ CM CD8$^+$ T cells**		F(2,24)= 3,765	**0,037**	0,225						F(2,24)= 7,199	**0,004**	0,375					
	Last relapse (months)				**0,126**	**0,048**	**0,457**	**2,619**	**0,015**				**0,200**	**0,053**	**0,616**	**3,774**	**0,001**
	Corticosteroids[1]				-0,433	1,030	-0,073	-0,420	0,678				0,206	1,164	0,029	0,177	0,861
**CCR4$^+$ EM CD8$^+$ T cells**		F(2,24)= 3,435	**0,047**	0,209													
	Last relapse (months)				**0,117**	**0,049**	**0,423**	**2,396**	**0,024**								
	Corticosteroids[1]				-0,727	1,045	-0,123	-0,695	0,493								
**CCR6$^+$ EM CD8$^+$ T cells**		F(2,24)= 6,926	**0,004**	0,348													
	Last relapse (months)				**-0,567**	**0,177**	**-0,514**	**-3,210**	**0,004**								
	Corticosteroids[1]				5,217	3,767	0,222	1,385	0,178								
**CCR10$^+$ EM CD8$^+$ T cells**		F(2,24)= 4,509	**0,021**	0,258						F(2,24)= 4,391	**0,024**	0,268					
	Last relapse (months)				**0,091**	**0,030**	**0,511**	**2,993**	**0,006**				**0,280**	**0,094**	**0,523**	**2,960**	**0,007**
	Corticosteroids[1]				0,128	0,650	0,034	0,197	0,845				1,209	2,072	0,103	0,584	0,565
**CCR10$^+$ TemRA CD8$^+$ T cells**		F(2,24)= 4,721	**0,018**	0,266													
	Last relapse (months)				**-0,022**	**0,010**	**-0,373**	**-2,196**	**0,037**								
	Corticosteroids[1]				0,377	0,210	0,305	1,798	0,084								
**Tregs**										F(2,24)= 7,401	**0,003**	*0,381*					
	Last relapse (months)												**2,215**	**0,615**	**0,585**	**3,602**	**0,001**
	Corticosteroids[1]												25,290	13,483	0,305	1,876	0,073

[1]Reference is "No history of Corticosteroids"; **CM**, Central memory; **EM**, Effector Memory; **NK**, Natural Killer cells; **NKT**, Natural Killer cells; **TemRA**, Terminally differentiated CD45RA-expressing memory cells; **Th**, Helper T cells; **Treg**, Regulatory T cells

eTable 10 (cont.)

Dependent Variable	Independent Variable	Percentage of cells								Absolute number of cells							
		Model	p-value	Effect Size (R^2)	B	SE	*β*	*t*	*p-value*	Model	p-value	Effect Size (R^2)	B	SE	*β*	*t*	*p-value*
**CD45RA$^+$HLA-DR$^-$ Tregs**										F(2,24)= 9,065	**0,001**	0,430					
	Last relapse (months)												**0,801**	**0,197**	**0,633**	**4,059**	**0,000**
	Corticosteroids[1]												8,119	4,326	0,292	1,877	0,073
**CD45RA$^-$HLA-DR$^-$ Tregs**										F(2,24)= 5,129	**0,014**	0,299					
	Last relapse (months)												**0,981**	**0,325**	**0,521**	**3,016**	**0,006**
	Corticosteroids[1]												10,819	7,131	0,262	1,517	0,142
**CD45RA$^-$HLA-DR$^+$ Tregs**										F(2,24)= 4,388	**0,024**	0,268					
	Last relapse (months)												**0,417**	**0,159**	**0,462**	**2,616**	**0,015**
	Corticosteroids[1]												6,170	3,496	0,312	1,765	0,090
**CD39$^+$ Tregs**										F(2,24)= 4,345	**0,025**	*0,266*					
	Last relapse (months)												**0,685**	**0,239**	**0,507**	**2,865**	**0,009**
	Corticosteroids[1]												5,838	5,244	0,197	1,113	0,277
**NK cells**										F(2,18)= 3,018	0,074	*0,251*					
	Last relapse (months)												**25,902**	**11,729**	**0,456**	**2,208**	**0,040**
	Corticosteroids[1]												112,313	156,787	0,148	0,716	0,483
**CD56^{dim}CD57$^+$ NK cells**										F(2,18)= 4,928	**0,020**	*0,354*					
	Last relapse (months)												**16,404**	**5,607**	**0,561**	**2,925**	**0,009**
	Corticosteroids[1]												49,904	74,957	0,128	0,666	0,514
**NKT cells**										F(2,18)= 6,033	**0,010**	*0,401*					
	Last relapse (months)												**10,494**	**3,092**	**0,627**	**3,394**	**0,003**
	Corticosteroids[1]												8,161	41,332	0,036	0,197	0,846

[1]Reference is "No history of Corticosteroids"; **CM**, Central memory; **EM**, Effector Memory; **NK**, Natural Killer cells; **NKT**, Natural Killer cells; **TemRA**, Terminally differentiated CD45RA-expressing memory cells; **Th**, Helper T cells; **Treg**, Regulatory T cells

eTable 11. Linear regression models to evaluate the impact of the Multuple Sclerosis Severity Score (MSSS) on the several blood cell populations. The unstandardized residuals from a preliminary multiple linear regression were calculated where the contribution of sex, age and HCMV IgG seroprevalence on the blood populations was evaluated. The residuals were used as dependent variable to evaluate the impact of MSSS of the blood cell populations. Only the blood populations for which the linear regressions were significant (p <0.050) are represented.

Dependent Variable	Independent Variable	Percentage of cells							Absolute number of cells						
		Model	Effect Size (R^2)	B	SE	*β*	*t*	*p-value*	Model	Effect Size (R^2)	B	SE	*β*	*t*	*p-value*
**sjTREC levels**									F(1,19)= 5,704	0,231					
	MSSS										**-69,351**	**29,039**	**-0,481**	**-2,388**	**0,027**
**Th17**		F(1,28)= 4,607	0,141						F(1,26)= 4,430	0,146					
	MSSS			**-0,113**	**0,053**	**-0,376**	**-2,146**	**0,041**			**-1,277**	**0,607**	**-0,382**	**-2,105**	**0,045**
**Naive/Activated Tregs**		F(1,28)= 6,840	0,196												
	MSSS			**-0,039**	**0,015**	**-0,443**	**-2,615**	**0,014**							
**CD45RA$^+$HLA-DR$^-$ Tregs**		F(1,28)= 6,783	0,195												
	MSSS			**-1,365**	**0,524**	**-0,442**	**-2,604**	**0,015**							
**GARP$^+$ Tregs**		F(1,28)= 4,997	0,151						F(1,26)= 4,679	0,153					
	MSSS			**0,722**	**0,323**	**0,389**	**2,235**	**0,034**			**0,875**	**0,404**	**0,391**	**2,163**	**0,040**
**KIR3DL1$^+$NKT cells**		F(1,23)= 6,434	0,219												
	MSSS			**1,408**	**0,555**	**0,468**	**2,537**	**0,018**							
**KIR2DL2/3$^+$ NKT cells**		F(1,23)= 5,818	0,202												
	MSSS			**0,661**	**0,274**	**0,449**	**2,412**	**0,024**							

CM, Central memory; **EM,** Effector memory; **MSSS,** Multiple Sclerosis Severity Score; **NKT**, Natural Killer cells; **Th**, Helper T cells; **TREC**, T cell receptor excision circles; **Treg**, Regulatory T cells

eTable 12. Demographic and clinical characterization of the cohort.

	**Newly diagnosed RRMS Patients (n = 30)**	**Healthy Controls (n = 33)**
Age (years). Mean [range]	33.7 [19;54]	33.7 [21;55][1]
Men. % (*n*)	36.7 (11)	36.4 (12)[2]
Age at MS onset (years). Median [range]	31 [18;53]	na
Time since MS diagnosis (months). Median [range]	1 [0;17]	na
Time from last relapse (months). Median [range]	7 [0;49]	na
MSSS. Median [range]	2.4 [0.7;9.4]	na
anti-HCMV IgG^+. % (*n*)	63.3 (19)	84.8 (28)[3]

[1] t_{61} = 0.000; $p > 0.9999$.

[2] χ^2 (df=1) = 0.001; $p = 0.980$.

[3] χ^2 (df=1) = 3.839; $p = 0.050$

HCMV, Human Cytomegalovirus; **MSSS**, Multiple Sclerosis Severity Score; **na**, not applicable

eTable 13. Panels of anti-human antibodies used for blood cells phenotypical characterization.

Panel	Target	Clone	Fluorochrome	Excitation laser	Detector	Dilution[1]	Company
**Recent Thymic Emigrants T cell homeostasis** (~1 million PBMCs)	CD19	HIB19	FITC	488	530/30	1:150	BioLegend
	CD3	OKT3	PE	488	575/26	1:200	BioLegend
	7AAD	na	na	488	695/40	1:40	BioLegend
	CD31	WM59	PE-Cy7	488	780/60	1:80	BioLegend
	CD8	RPA-T8	APC	633	660/20	1:150	BioLegend
	CD4	RPA-T4	APC-Cy7	633	780/60	1:80	BioLegend
	CD45RA	HI100	Pacific Blue	407	450/50	1:300	BioLegend
	CD45	HI30	BV510	407	525/50	1:80	BioLegend
	CD45RO	UCHL1	BV650	407	660/20	1:300	BioLegend
	CCR7	G043H7	BV785	407	780/60	1:40	BioLegend
**$CD4^+$ and $CD8^+$ T cell homeostasis** (~1 million PBMCs)	CD3	UCHT1	FITC	488	530/30	1:25	BD Pharmingen
	CCR10	1B5	PE	488	575/26	1:1600	BD Pharmingen
	CD45RA	HI100	PercP-Cy5.5	488	695/40	1:80	BioLegend
	CD56	HCD56	PE-Cy7	488	780/60	1:80	BioLegend
	Fixable Viability dye	na	eFluor660	633	660/20	1:1000	eBioscience
	CD4	RPA-T4	APC-Cy7	633	780/60	1:80	BioLegend
	CCR4	L291H4	BV421	407	450/50	1:80	BioLegend
	CD45	HI30	BV510	407	525/50	1:80	BioLegend
	CCR6	G034E3	BV650	407	660/20	1:80	BioLegend
	CxCR3	G025H7	BV711	407	710/50	1:80	BioLegend
	CCR7	G043H7	BV785	407	780/60	1:40	BioLegend
**Regulatory T cells (Tregs)** (~1 million PBMCs)	CD39	A1	FITC	488	530/30	1:80	BioLegend
	FoxP3	PCH101	PE	488	575/26	1:20	eBioscience
	CD45RA	HI100	PercP-Cy5.5	488	695/40	1:80	BD Pharmigen
	HLA-DR	L243	PE-Cy7	488	780/60	1:30	BioLegend
	GARP	7B11	APC	633	660/20	1:80	eBioscience
	CD4	RPA-T4	APC-Cy7	633	780/60	1:80	BioLegend
	CD25	BC96	BV421	407	450/50	1:20	BioLegend
	Fixable Viability dye	na	BV510	407	525/50	1:1000	BioLegend
	CD45	HI30	BV605	407	610/20	1:40	BioLegend
	CD3	OKT3	BV650	407	660/20	1:40	BioLegend
	CD127	A019D5	BV711	407	710/50	1:20	BioLegend
	CD73	AD2	BV785	407	780/60	1:80	BioLegend
**Inhibitory Receptors NK/NKT cells** (~1 million PBMCs)	NKG2A	REA110	VioBright FITC	488	530/30	1:100	MACS Miltenyi
	KIR2DL2/3	DX27	PercP-Cy5.5	488	695/40	3:50	BioLegend
	CD57	HNK-1	PE-Cy7	488	780/60	1:25	BioLegend
	Fixable Viability dye	na	eFluor660	633	660/20	1:1000	eBioscience
	KIR3DL1	DX9	APC-Fire750	633	780/60	1:25	BioLegend
	KLRG1	14C2A07	BV421	407	450/50	3:50	BioLegend
	CD3	UCHT1	V500	407	525/50	1:25	BD Horizon
	CD45	HI30	BV605	407	610/20	1:25	BioLegend
	CD27	L128	BV650	407	660/20	1:25	BD Horizon
	CD56	NCAM16.2	BV786	407	780/60	1:25	BD Horizon

[1] In a final volume of 100 µL; na, not applicable

eTable 13 (cont.)

Panel	Target	Clone	Fluorochrome	Excitation laser	Detector	Dilution[1]	Company
**Activating Receptors NK/NKT cells** (~1 million PBMCs)	NKp44	P44-8	PE	488	575/26	1:25	BioLegend
	NKp46	.9E2	PerCP eFluor711	488	695/40	1:50	eBioscience
	CD57	HNK-1	PE-Cy7	488	780/60	1:25	BioLegend
	Fixable Viability dye	na	eFluor660	633	660/20	1:1000	eBioscience
	NKG2D	1D11	APC-Cy7	633	780/60	1:25	BioLegend
	CD3	UCHT1	V500	407	525/50	1:25	BD Horizon
	CD45	HI30	BV605	407	610/20	1:25	BioLegend
	CD27	L128	BV650	407	660/20	1:25	BD Horizon
	NKp30	P30-15	BV711	407	710/50	1:25	BioLegend
	CD56	NCAM16.2	BV786	407	780/60	1:25	BD Horizon

[1] In a final volume of 100 µL; na, not applicable

eTable 14. Single joint (sj) and DbetaJbeta (DJβ) T cell receptor excision circles (TRECs) and CD3 primers sequences and quantity used in the nested-PCR.

Primer		Sequence (5'-3')	Final concentration	Company
sjTRECs	OUT 3’	ACATTTGCTCCGTGGTCTGT	1 µM	STABVIDA
	OUT 5’	CTCTCCTATCTCTGCTCTGAA	1 µM	STABVIDA
	IN 3’	GTGCTGGCATCAGAGTGTGT	1,4 µM	STABVIDA
	IN 5’	TGATGCCACATCCCTTTCAA	1,4 µM	STABVIDA
DJβTREC	OUT 3'	CTCATCTGGGCCTGTCCTTGT	1 µM	STABVIDA
	IN 3’	TGACCCAGGAGGAAAGAAG	0,28 µM	STABVIDA
	1.1 - OUT 5'	AACCTAGGACCCTGTGGATG	1 µM	STABVIDA
	1.1 - IN 5'	TGTCCTCCATCCTAGCCAGG	0,28 µM	STABVIDA
	1.2 - OUT 5'	CTCTCTATGCCTTCAATGTG	1 µM	STABVIDA
	1.2 - IN 5'	TCCGTCACAGGGAAAAGTGG	0,28 µM	STABVIDA
	1.3 - OUT 5'	AAGGGAACACAGAGTACTGGAA	1 µM	STABVIDA
	1.3 - IN 5'	TCCCAACCTCTGCCTGAAT	0,28 µM	STABVIDA
	1.4 - OUT 5'	TGGACTTGGGGAGGCAGGA	1 µM	STABVIDA
	1.4 - IN 5'	AGGAGTGGAAGGCAGCAGGT	0,28 µM	STABVIDA
	1.5 - OUT 5'	GAAACTGAGAACACAGCCAAGAA	1 µM	STABVIDA
	1.5 - IN 5'	CTCATAAAATGTGGGTCAGTGGA	0,28 µM	STABVIDA
	1.6 - OUT 5'	ATCCTCCCTCTTATGTGCATGG	1 µM	STABVIDA
	1.6 - IN 5'	TGAATCCAGGCAGAGAAAGG	0,28 µM	STABVIDA
CD3γ	OUT 3’	AGCTCTGAAGTAGGGAACATAT	1 µM	STABVIDA
	OUT 5’	ACTGACATGGAACAGGGGAA	1 µM	STABVIDA
	IN 3’	CCTCTCTTCAGCCATTTAAGTA	1,4 µM	STABVIDA
	IN 5’	GGCTATCATTCTTCTTCAAGGTA	1,4 µM	STABVIDA
